# A Bayesian Inference Approach to Accurately Fitting the Glass Transition Temperature in Thin Polymer Films

**DOI:** 10.1021/acs.macromol.4c01867

**Published:** 2024-11-22

**Authors:** James H. Merrill, Yixuan Han, Connie B. Roth

**Affiliations:** Department of Physics, Emory University, Atlanta, Georgia 30322, United States

## Abstract

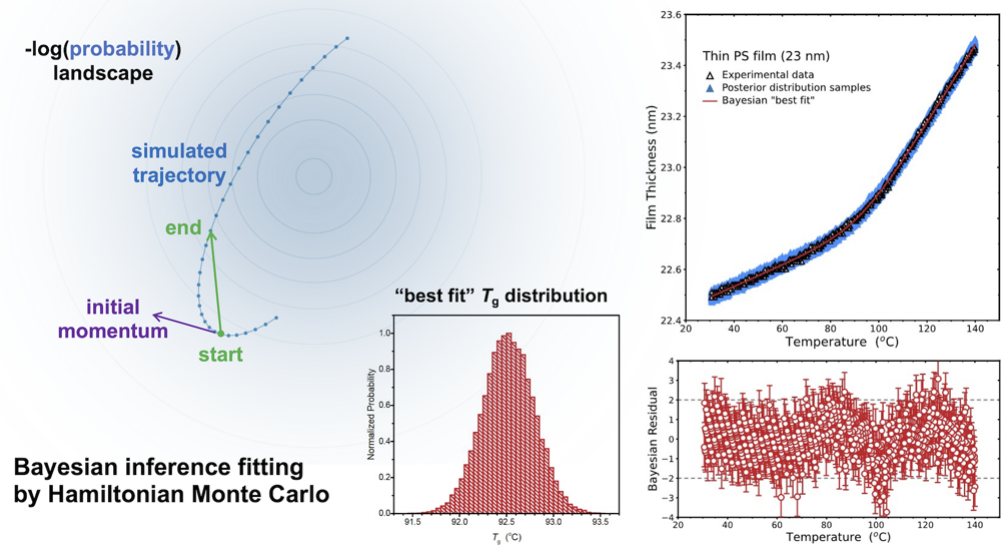

We present a Bayesian inference-based nonlinear least-squares fitting approach developed to reliably fit challenging, noisy data in an automated and robust manner. The advantages of using Bayesian inference for nonlinear fitting are demonstrated by applying this approach to a set of temperature-dependent film thickness *h*(*T*) data collected by ellipsometry for thin films of polystyrene (PS) and poly(2-vinylpyridine) (P2VP). The glass transition experimentally presents as a continuous transition in thickness characterized by a change in slope that in thin films with broadened transitions can become particularly subtle and challenging to fit. This Bayesian fitting approach is implemented using existing open-source Python libraries that make these powerful methods accessible with desktop computers. We show how this Bayesian approach is more versatile and robust than existing methods by comparing it to common fitting methods currently used in the polymer science literature for identifying *T*_g_. As Bayesian inference allows for fitting to more complex models than existing methods in the literature do, our discussion includes an in-depth evaluation of the best functional form for capturing the behavior of *h*(*T*) data with temperature-dependent changes in thermal expansivity. This Bayesian fitting approach is easily automated, capable of reliably fitting noisy and challenging data in an unsupervised manner, and ideal for machine learning approaches to materials development.

## 1 Introduction

Accurate experimental determination of the glass transition temperature *T*_g_ of thin polymer films is important both for developing first-principles understanding of the physics of confined nanoscale materials and for improving applications such as dielectric and adhesive coatings, nanolithography, and separation membranes.^1−4^ The glass transition manifests as a second-order phase transition being discontinuous in the second partial derivatives of the free energy.^5^ However, being a kinetic transition, the glass transition temperature depends on the rate at which it is measured. Experimentally, the glass transition is frequently identified from the continuous transition in first-order thermodynamic parameters such as enthalpy *H* and volume *V*. Correctly identifying *T*_g_, along with a good estimate of its error, from such continuous data was already identified as a nontrivial task over half a century ago,^6^ and remains so today. For thin films, the film thickness *h* acts as a proxy for volume.^7−9^ Consequently, the challenge with identifying *T*_g_ is that the small size of the sample typically leads to low signal-to-noise ratio, making accurate determination of *T*_g_ problematic.^10^ The sharpness of the glass transition can be further compromised in thin films by an increase of the transition width that often signifies a gradient in local dynamics with depth due to perturbations from the free surface or substrate interface.^11,12^ As a result, these factors make identifying *T*_g_ of very thin films challenging. The growth of machine learning and automation to accelerate polymer characterization and materials development requires that data analysis becomes more unsupervised, while still remaining robust and reliable.^13−15^ We present here a Bayesian inference-based nonlinear least-squares fitting approach that can be reliably applied in an automated manner to fit challenging data in order to reliably identify the glass transition in thin polymer films. With the Python code provided, this Bayesian fitting approach can be easily adapted to fit other challenging data sets with any appropriate user-defined functional form.

Temperature-dependent film thickness data *h*(*T*) for thin polymer films, as can be collected by ellipsometry^16−20^ or X-ray reflectivity,^21^ exhibits two linear regions above and below *T*_g_ corresponding to the thermal expansion of the material. Most commonly *T*_g_ is identified from the intersection of two linear fits done to the *h*(*T*) data above and below *T*_g_. Data by other experimental techniques such as fluorescence^12,22−24^ also frequently identify *T*_g_ by a similar intersection of two linear fits. While simple, the fitting of two linear regions above and below *T*_g_ involves more user/scientist input than is often acknowledged. Not only does a decision need to be made on the overall initial temperature range that the data is collected over, but also then the range of temperatures above and below *T*_g_ over which the linear fits are made (i.e., the fitting windows). In particular, for thin films that show a broadened transition compared to bulk, some region of the data around the transition frequently needs to be excluded, where small changes in these fitting window ranges can strongly impact the *T*_g_ value obtained. Some previous studies have highlighted the importance of collecting data over a sufficiently wide temperature range,^12,19^ or have attempted to systematize the process of identifying the best linear fits by, for example, maximizing statistical quantities like *R*^2^.^22,25,26^ Other studies have emphasized the value in defining a consistent process for identifying *T*_g_ from linear fits, suggesting possible temperatures where tangent lines should be drawn.^27,28^ Alternatively, some efforts have been made to examine the breadth of the transition in more detail by numerically differentiating the *h*(*T*) data,^11,12,17,29^ but such analyses require further decisions about the Δ*T* range of the finite difference^11^ and/or local smoothing, as numerical differentiation accentuates the noise in the data.^12^

To address some of these issues, Dalnoki-Veress et al.^18^ introduced an empirical fitting function for temperature-dependent film thickness data by ellipsometry:
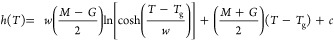
1This equation is derived by integrating an empirical expression for the thermal expansion coefficient  that transitions smoothly from a liquid state slope  to a glassy state slope  over a finite temperature range *w* centered on *T*_g_, assuming the profile of a hyperbolic tangent:
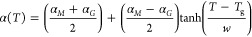
2The constant *c* of integration in eq 1 corresponds to the value of the film thickness at *T*_g_: *h*(*T*_g_). Use of eq 1 to determine *T*_g_ from *h*(*T*) or other similar data has become popular as it avoids needing to identify specific fitting windows for the linear fits above and below *T*_g_, while also fitting the data through the glass transition itself.^10,19,30−36^ Dalnoki-Veress et al. initially applied this equation to ellipsometry data on free-standing polystyrene (PS) films that exhibit a particularly sharp transition, where they chose to keep the width parameter *w* fixed at 2 °C.^18^ In contrast, use of eq 1 to fit supported polymer films necessitates keeping the width *w* as a variable fit parameter or fixed at a larger value, as the breadth of the glass transition is larger and increases with decreasing film thickness.^32,33,35^ Use of eq 1, compared to doing two linear fits, removes two implicit fit parameters that correspond to the range of data to exclude around the transition and replaces it with a single explicit parameter for the transition width *w*.^18^ However, care must still be taken to collect data over a sufficiently large temperature range to ensure that the liquid and glass slopes are accurately determined because eq 1 can suffer from the same problem as using the intersection of two linear fits, where both methods will identify *T*_g_ as simply the middle of a short data set with a broad transition that may appear uniformly curved.

Fitting to eq 1 requires the use of nonlinear least-squares methods, which can have a number of drawbacks and limitations. The most commonly used is the Levenberg–Marquardt (LM) algorithm, which identifies the nearest minimum in χ^2^ from the initial parameter guesses by first doing a gradient descent followed by a local parabolic expansion about the minimum.^37,38^ However, there is no guarantee that this process will find the global minimum and it is prone to getting stuck in local minima depending on the initial guesses for the parameter values. Fitting eq 1 to *h*(*T*) data is particularly sensitive to the initial conditions of the fit parameters, especially if the width *w* is variable. In our experience, we find that the initial values of the fit parameters need to be set quite close to the global minimum in χ^2^ for it to be accurately identified.

To address this, here we apply Bayesian inference to agnostically determine the probability distribution for each parameter in eq 1 when fit to *h*(*T*) data for supported PS films collected by ellipsometry. In particular, we use a Hamiltonian Monte Carlo algorithm to sample the likelihood function that defines the probability of an experimental *h*(*T*) data set having been generated by a given set of fit parameters. This takes advantage of modern computational tools that have been developed for statistical inference, and applied to computational optimization problems with multiple solutions and many parameters in the field of machine learning.^39−41^ Specifically, we leverage the existing open-source library PyMC in Python^42^ to create a statistical model that represents the data generation process and perform the optimization of the likelihood function. This method is applied to *h*(*T*) data for supported PS films over a wide range of film thicknesses *h* from 650 nm down to 21 nm resulting in best-fit probability distributions for *T*_g_(*h*), as well as the other fit parameters, notably *w*(*h*). We compare *T*_g_(*h*) trends and errors for this Bayesian inference method to the standard LM nonlinear fitting of eq 1, as well as the commonly used method of determining *T*_g_ by the intersection of two linear fits. Beyond the common user/scientist’s best determination of *T*_g_ by linear fits, we also computationally evaluate many possible linear fits in an exhaustive brute-force search to identify a distribution of *T*_g_ values from the intersections of these linear fits. We find that *T*_g_(*h*) is identified with greater precision by the nonlinear fit methods to eq 1. The Bayesian inference method requires less user input to arrive at the best-fit solution than the standard LM optimization that demands an initial guess already close to the global minimum. The Bayesian inference method also allows one to augment eq 1 by adding a nonlinear term to the glassy-state *h*(*T*) line to account for changes in thermal expansion of the glassy state with temperature not captured by the model.^43^ Although commonly done, we find that overall determination of *T*_g_ by the intersection of two linear fits results in larger errors, especially for thin films, where it is reasonable to conclude that some of the variation in *T*_g_(*h*) observed in the literature^44^ for thin films could result from user/scientist choices in their selection of fitting window and/or data range. We also compare values of the transition width *w* as a function of film thickness, demonstrating a broadening of the glass transition in supported films that starts at a similar critical thickness to *T*_g_(*h*).

## 2 Experimental Methods

All data plotted in this study were originally collected and published in two previous studies from our lab investigating the refractive index of thin polymer films.^20,45^ In particular, we use the data for polystyrene (PS) with *M*_w_ = 650 kg/mol, *M*_w_/*M*_n_ = 1.06 from Pressure Chemical, and poly(2-vinylpyridine) (P2VP) with *M*_w_ = 650 kg/mol, *M*_w_/*M*_n_ = 1.08 from Scientific Polymer Products and *M*_w_ = 643 kg/mol, *M*_w_/*M*_n_ = 1.18 from Polymer Source. Films of different thicknesses were made by spin-coating onto 2 cm × 2 cm silicon pieces at varying spin speed and solution concentration by dissolving PS in toluene or P2VP in butanol. To drive off any residual solvent and relax chain conformations, films were annealed for 12 h at *T*_g_^bulk^ + 25 °C under vacuum. Immediately prior to the measurement, samples were held at *T*_g_^bulk^ + 45 °C for 20 min on the ellipsometer heater stage to erase thermal history and equilibrate the polymer films.

Spectroscopic ellipsometry was used to determine the film thickness *h*(*T*) and refractive index *n*(*T*) as a function of temperature, where the data were collected on cooling at 1 K/min. Measurements of the ellipsometric angles Ψ(λ) and Δ(λ) were collected at an angle of incidence of 65°, over a wavelength range spanning λ = 400–1000 nm. Ψ(λ) and Δ(λ) data were fit to an optical layer model with a homogeneous Cauchy layer used to represent the polymer film:
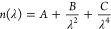
3where the Cauchy coefficient *C* was held constant at the value for bulk PS.^20,45^ The silicon substrate was modeled as a semi-infinite substrate with a 1.25 nm native oxide layer with all the associated optical constants held at literature values in the Woollam software.^20,45^ Thus, the parameters fit for the polymer layer are the film thickness *h*, and the Cauchy *A* and *B* parameters.

The nonlinear fit to the optical layer model generates uncertainties in the *h*(*T*) data resulting from the ellipsometry measurement uncertainties in Ψ and Δ. In our analysis of the *h*(*T*) data, we retain the root-mean-square (RMS) fitting error on *h* provided by the Woollam software (square-root of the diagonal covariance matrix element) as an estimate of the measurement error. The Woollam software also provides Monte Carlo and sensitivity analysis tools for estimating the uncertainty in the fitting parameters of the optical layer model. These tools both identified errors smaller than the RMS fitting error, so we can be reasonably sure we are not underestimating the error in *h*. This error in *h* is then used to weight the contribution of each data point in the sum of squared residuals during the fitting of *h*(*T*). Similarly, errors for bulk *T*_g_ from each fitting method were determined by taking an error-weighted average of the *T*_g_ values for films with thicknesses *h* > 100 nm. It is worth noting that at times the optical layer model fitting can be nontrivial, where a Bayesian approach to fitting has been shown to be helpful in finding a unique optimal solution when standard fitting techniques become difficult.^46^

## 3 Results and Discussion

Our main goal is a detailed analysis of fitting temperature-dependent film thickness data *h*(*T*) for supported PS thin films collected by ellipsometry^20,45^ to accurately identify the glass transition temperature *T*_g_ and its associated error. In particular, we compare the two main existing methods commonly used in the field to our proposed Bayesian analysis approach where no predetermined initial estimates of parameters need to be provided. For comparison purposes, we will illustrate the fitting results for two representative data sets: one from a 160 nm (bulk-like) PS film and one collected from a 23 nm (thin) PS film with a nominally reduced *T*_g_. In each subsection below, we outline the implementation of the fitting methods, apply them, then analyze the relative merits of each approach.

We start by contrasting the two existing methods commonly used in the field: (1) separately fitting two straight lines above and below the transition where *T*_g_ is identified from the intersection of those fits; and (2) doing a nonlinear least-squares fit of eq 1 to the full *h*(*T*) data set. We highlight how the error in *T*_g_ is accurately identified in each case. As much of our focus will be on the use of eq 1 as a means to fit the *h*(*T*) data, we next examine how accurately eq 1 captures the change in slope of the *h*(*T*) data through the glass transition by inspecting how eq 2 fits the temperature dependence of the thermal expansivity  obtained by numerically differentiating the *h*(*T*) data. Although we find that eq 2 may not produce the most ideal description of the α(*T*) transition, especially for thin films, use of eq 1 is still the most feasible analytical equation to fit the *h*(*T*) data.

Then we describe in detail the proposed Bayesian inference fitting approach employed to identify *T*_g_ and associated error from *h*(*T*) data using eq 1. Fitting is accomplished by maximizing a likelihood function that is constructed from the sum of squared residuals between the experimental *h*(*T*) data and the *h*(*T*) model of eq 1, where a Hamiltonian Monte Carlo routine is used to efficiently sample the likelihood function at different parameter values. One key advantage of a Bayesian approach is that each parameter is represented by a probability distribution. This means that the initial distribution for a given parameter can easily be defined as uniform, and the final “best-fit” parameter values are determined agnostically from the resulting “best-fit” probability distributions. We demonstrate that a Bayesian approach can accommodate more complex models for the *h*(*T*) data and can be easily implemented and readily fit. This is in contrast to standard nonlinear fitting approaches where eq 1 is already at the limit of complexity to obtain reasonable fits.

We also examine the variability in possible *T*_g_ fit values obtained by the linear-fit intersection method by using an exhaustive approach that evenly samples over a large range of all the possible choices of fitting windows according to some simple rules. This mimics the variability that might be observed in the literature across different researchers, and allows us to plot histograms of the relative frequency with which certain *T*_g_ values might be obtained for a given data set. An idealized data set is used to validate this approach and ensure that the obtained distribution is peaked at the “true” *T*_g_ value. Applying this exhaustive search to experimental data sets, we find that a wide range of *T*_g_ values are possible, varying greatly with fitting window selection, where the breadth of the distribution of possible *T*_g_ values is much larger than the fitting error obtained for *T*_g_ from a single set of linear fits.

Finally, we examine the film thickness dependence of *T*_g_(*h*), as well as the transition width *w*(*h*), for an extensive collection of *h*(*T*) data sets spanning a range of film thicknesses. We find that the *T*_g_(*h*) values obtained from all fitting methods overlap remarkably well, albeit with slightly different absolute values of *T*_g_^bulk^. The nonlinear methods (standard LM and Bayesian) give a consistently smaller error on individual *T*_g_ values than that obtained by determining the intersection of linear fits. We note that this is especially true given that there is typically additional (unknown) error associated with the determination of *T*_g_ from linear fits due to the requirement of selecting fitting windows, as illustrated by the brute-force method. From the Bayesian analysis, we find that the transition width *w*(*h*) is constant for bulk-like samples, but increases for thin films below a critical thickness ≤45 nm that is similar to the critical thickness below which *T*_g_(*h*) itself decreases. For ultrathin films *h* < 20 nm, the Bayesian fitting method is able to determine *T*_g_ with considerably less error than the commonly employed method of identifying the intersection of two linear fits. Interestingly, transition width *w*(*h*) from the Bayesian fits shows a reduction in glass transition breadth for ultrathin films likely reflecting a narrowing of the distribution of relaxation times for *h* < 20 nm. We also apply the Bayesian fitting method to thin films of P2VP.

### 3.1 Common Method of Identifying *T*_g_ via Intersection of Two Linear Fits

**Figure 1:**
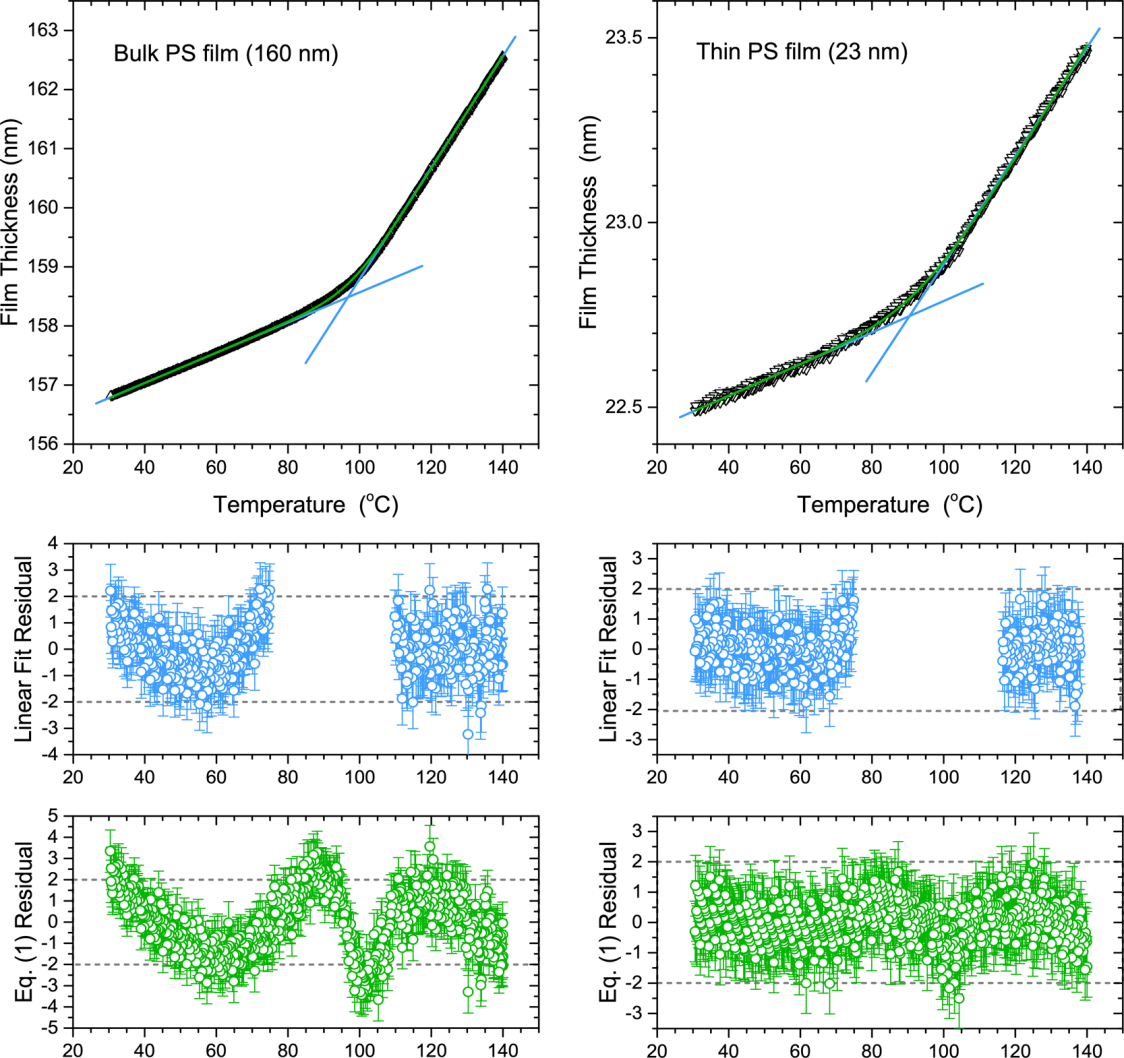
Top panels: Temperature-dependent film thickness measured on cooling, for a bulk-like 160 nm PS film on the left and for a thin 23 nm film on the right. Black triangles are the measured film thickness values, while blue lines are linear fits of the liquid and glassy states, and the green curve is the best fit to eq 1 using standard Levenberg–Marquardt (LM) optimization. Lower panels are studentized residuals for the linear fits shown in blue and LM fits to eq 1 in green, where the *y*-axis is scaled by the error σ of the *h*(*T*) data, with the range of −2σ to 2σ marked by gray dashed lines to highlight where 95% of the data should fall. Residuals are reasonably well-behaved, although the data for the thick film are sufficiently precise to observe systematic curvature, particularly in the glassy state, while this is in the noise for the thin film.

The most common approach used in the literature for determining the glass transition temperature *T*_g_ from experimental *h*(*T*) data is to perform linear fits to two subsets of the data representing the glassy and liquid temperature regions, where *T*_g_ is then identified from the intersection of these two linear fits. In Figure 1, we show the temperature-dependent film thickness *h*(*T*) data for the two representative PS films: 160 nm (bulk-like) and 23 nm (thin film). As is usually done by researchers, the linear fits were done to the glassy and liquid states by selecting temperature regions that were sufficiently far from the transition region to not be biased by the changing *h*(*T*) slope. This excluded a range of data spanning approximately 25 °C about the presumed transition temperature. The value for *T*_g_ is then determined from the intersection of the two linear fits (*m*_1_*T* + *b*_1_ and *m*_2_*T* + *b*_2_) by simple algebra:
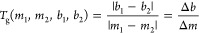
4giving 96.5 °C for the 160 nm thick film and 90.6 °C for the 23 nm thin film.

The uncertainty in the derived *T*_g_ value can be calculated by simple error propagation of eq 4 as
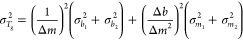
5The σ values correspond to the variance in each of the subscripted fit parameters, determined from the diagonal components of the covariance matrix, which are only physically meaningful if the errors on *h* are known and carried through all the calculations. Equation 5 gives an uncertainty of ±0.2 °C for the 160 nm thick film and ±2 °C for the 23 nm thin film. Defining an error in *T*_g_ based on these fit statistics is often overlooked in the field, as the fitting errors can be (but are not always) small compared to the variation from sample-to-sample, particularly for thick films where the data have relatively low noise. We note that eq 5 assumes uniform errors in *h*, where Filliben and McKinney addressed the more complicated calculation to obtain the error in *T*_g_ for the case of nonuniform errors in the data from pressure-dependent viscosity data.^6^ For the ellipsometric data analyzed here, the error in *h* is reasonably approximated as uniform.

To evaluate the quality of these fits, Figure 1 also plots the studentized residuals, calculated by taking the predicted (fit) *h* values minus the experimentally observed values, and dividing by the measurement error in *h* (obtained from the ellipsometric fits to the optical layer model). For a “good fit” there should be no obvious patterns in the residuals, and approximately 95% of the points should fall within two times the measurement error of the fit, as regression analysis assumes normally distributed errors. From the linear fit residual shown in Figure 1, the linear fits may be considered good as most of the points lie within 2 errors in *h* of the fit. However, there is some curvature in the glassy state data, more noticeable for the bulk film compared to the thin film due to the greater precision in *h* relative to the measured value, suggesting a small systematic deviation from the fit line in the glassy state that may be worth accounting for by changing the functional form being fit to.

The major issue with this method of determining *T*_g_ from the intersection of two linear fits is that there is considerable variability associated with the selection of the data range used for the linear fits that is difficult to quantify. In Section 3.5, we will examine how the choice of fitting window impacts the *T*_g_ value determined from a given data set. In the following section, we consider another way of avoiding the ambiguity of the linear-fit-intersection method by using a nonlinear fitting approach where all the data can be included in the fit.

### 3.2 Nonlinear Fitting to Eq 1

Using nonlinear least-squares methods with an appropriate functional form to fit the data is an attractive option due to the lack of ambiguity in the *T*_g_ value obtained, and the ability to include all of the data through the transition region. The functional form in eq 1 was originally proposed by Dalnoki-Veress et al.^18^ to fit ellipsometric film thickness data *h*(*T*) from free-standing polymer films. Five explicit fit parameters appear in eq 1: *T*_g_, *c* = *h*(*T*_g_), slopes *M* and *G*, plus the transition width *w*. Although the linear-fit-intersection method appears to only have four fit parameters (*m*_1_, *m*_2_, *b*_1_, *b*_2_ in eq 4), there are actually at least two additional implicit fit parameters resulting from the selection of the temperature boundaries, notably the two inner fitting window boundaries that demarcate what data is being excluded around the transition region. We note that in the original study on free-standing films, the transition width *w* was held fixed at 2 °C by Dalnoki-Veress et al. (further eliminating a fit parameter) because free-standing films exhibit sharp transitions for all film thicknesses.^18^ However, when applying the same functional form to fit data for supported films, especially very thin films, a single, constant value of transition width *w* is not sufficient to obtain acceptable quality fits for a wide range of film thicknesses as the breadth of the glass transition increases with decreasing film thickness.

Figure 1 shows the best fit curves and residuals from fitting eq 1 to the *h*(*T*) data employing the commonly used Levenberg–Marquardt (LM) algorithm. The LM algorithm interpolates between a gradient descent approach and an inverse Hessian, i.e., a second-order series expansion about the minimum, to iteratively minimize χ^2^.^37^ For the two representative data sets shown in Figure 1, the fits to eq 1 are good, giving *T*_g_ = 97.60 ± 0.04 °C for the 160 nm thick film and *T*_g_ = 92.8 ± 0.4 °C for the 23 nm thin film. The best-fit values of the transition width *w* are 16.5 °C for the thin film and 10.2 °C for the thick film, quantifying the substantial increase in transition width with decreasing film thickness. These errors represent the fitting error identified from the root-mean-square error of the relevant fit parameter (diagonal covariance matrix element).

Similar to the linear-fit-intersection method, one can identify some systematic curvature in the residual in Figure 1, especially in the glassy regime for the bulk film, indicating some nonlinear expansion with temperature. Some curvature appears in the liquid state residual too, but it is more subtle and could be due to the model not fully capturing the behavior in the transition region, a possibility discussed further in the following section. One could argue that due to the number of points that lie outside the ±2 error threshold in *h*, the fit to eq 1 is “worse” than the linear fits. This observation can reasonably be explained by the fact that the linear fit approach makes no attempt to model *h*(*T*) in the transition region, and therefore the choice of inner temperature boundaries on the fitting windows nearly entirely determines the quality of the fits. Fitting to a shorter region of data will improve linearity (and thus fit quality by this metric) at the expense of precision in the best-fit parameter values in general.

These fits show that using the nonlinear fitting approach works for supported PS films, and identifies an unambiguous *T*_g_. However, the main drawback with the LM algorithm and other iterative least-squares optimization algorithms is the tendency to yield solutions not corresponding to the global minimum in χ^2^ if the initial values for the parameters are not already somewhat close to the optimal solution.^37,47^ A guess for the initial conditions is always necessary, and can be challenging if the various parameter values differ by many orders of magnitude. For example, initial values for the thermal expansivities α_*M*_ and α_*G*_, which are 6 orders of magnitude smaller than *T*_g_, can be estimated by quick linear fits to the liquid and glassy portions of the *h*(*T*) data. One inconvenience resulting from this is that these fits cannot easily be automated across data sets without a human inputting initial parameter values for each fit. In addition, the requirement that a human supply these initial values could introduce some degree of bias in the fits that is difficult to quantify. In fitting eq 1 using the LM algorithm to the set of *h*(*T*) data over a range of different film thicknesses, we found that substantial hand-tuning of initial values was required to obtain the global minimum in χ^2^. By this we mean that a stable and robust minimum is reached that is invariant to small tweaks in initial parameter values. Given that this level of user-input is required to fit the five independent parameters of eq 1, adding more parameters to this functional form to account for some nonlinear thermal expansion seems potentially problematic using this fitting approach. This sets the stage for our efforts to find a better way of fitting *h*(*T*) data to the model eq 1 represents. Before going further though, we must first critically evaluate whether this model is the best one to use for temperature-dependent film thickness data.

### 3.3 Functional Form of Temperature-Dependent Thermal Expansion Coefficient α(*T*)

Equation 1, frequently used in the field to fit the glass transition in thin films, especially *h*(*T*) data measured by ellipsometry, is derived from the expression for the thermal expansion α(*T*) shown in eq 2. This expression assumes the average thermal expansion of the film is symmetric about the transition, varying smoothly from the glassy to liquid state expansion values as a hyperbolic tangent over some finite temperature range. Given that the *h*(*T*) functional form used for the nonlinear fitting is derived from an expression of the thermal expansivity α(*T*), some examination of what functional form fits α(*T*) data best is warranted.

**Figure 2:**
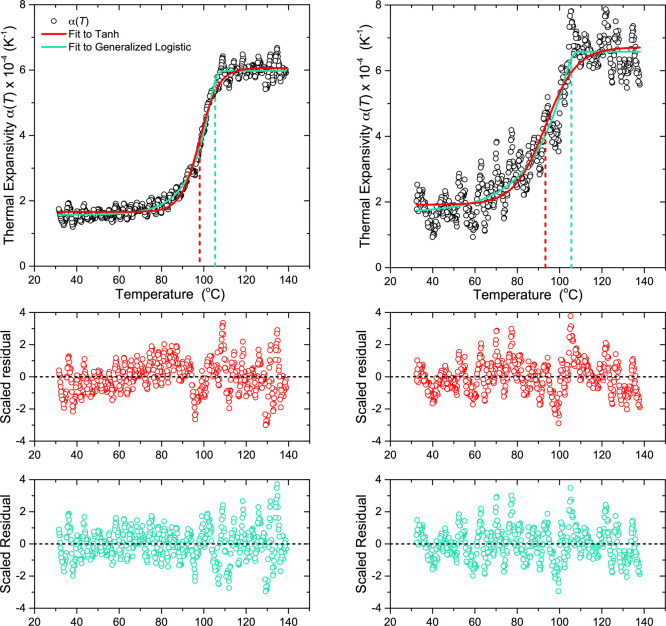
Top panel: Plots of the temperature-dependent thermal expansivity α(*T*) obtained by numerically differentiating the *h*(*T*) representative data sets for the 160 nm thick and 23 nm thin films. Data shown as black circles with the fit to the hyperbolic tangent eq 2 shown in red and the fit to the generalized logistic function eq 7 shown in turquoise, where the drop lines identify the *T*_g_ parameter. Lower panels are studentized residuals for the hyperbolic tangent shown in red and the generalized logistic shown in turquoise. The residuals for the tanh function exhibit clear systematic patterns with large deviations around the transition temperature itself and nonzero slope in the glassy state. In contrast, the residuals for the generalized logistic function show excellent fits with uniform noise about zero for both the bulk and thin film.

However, we need to recognize that α(*T*) is typically obtained by numerical differentiation of *h*(*T*) data, requiring a transformation of the data which has a number of possible approaches. The most straightforward is a simple finite-difference numerical derivative, as was done for supported PS films by Kawana and Jones,^11^ using a somewhat arbitrarily chosen 4.2 °C temperature interval for the finite difference. Here, we use a slightly more complicated procedure, performing linear fits on successive subsets of the data of length Δ*T* to determine a local slope at the center of the temperature interval. This procedure essentially yields a numerical derivative that is smoothed over a bandwidth of Δ*T*, with equal weighting over the temperature interval. The results of this transformation procedure are plotted in Figure 2 as the black data. A variety of temperature intervals were tested, with the best values selected being 2.5 °C for the bulk film and 4.2 °C for the thin film, producing a reasonable balance of smoothing out some of the noise while retaining the features present in the data.

Having settled on at least one reasonable method for differentiating the film thickness data, we now examine what the best functional form for fitting α(*T*) data might be. Kawana and Jones chose to fit three straight lines to the α(*T*) data, in each of the three regimes: liquid state, glassy state, and transition region.^11^ They chose to identify the intersection of the liquid line with the transition line as an “onset” temperature *T*_+_ on cooling and the intersection of the transition line with the glassy line as an “end point” temperature *T*_–_, with the midpoint between these two temperatures taken to be *T*_g_. They also observed that *T*_+_ stays relatively invariant with decreasing film thickness, while *T*_–_ shifts lower, indicating that the transition broadens asymmetrically in thin films with *T*_–_ exhibiting the largest change with decreasing film thickness.^11^ From the α(*T*) data in Figure 2, we clearly observe a similar behavior to that seen by Kawana and Jones, where the transition in the thin film data is noticeably asymmetric. The three-line construction used by Kawana and Jones is not a continuous function that can be easily integrated to give a functional form for *h*(*T*), and it is unable to capture the varying curvature at the glassy and liquid ends of the transition.

The tanh-based α(*T*) functional form of eq 2 provides a continuous function that can be easily integrated to arrive at the *h*(*T*) functional form of eq 1. However, by definition the tanh function is symmetric about the transition. In Figure 2, we plot the best fit tanh-based α(*T*) curve to the thick and thin film data, along with its residual. The *T*_g_ values obtained by fitting the numerically differentiated α(*T*) data are similar to those obtained from fitting the *h*(*T*) data directly with eq 1, although interestingly they are both 0.5 °C higher for the thick and thin films, *T*_g_ = 98.1 and 93.3 °C, respectively. We find that this tanh-based model does a reasonable job of describing the transition, but is not capturing the sharpness of the transition out of the liquid state and the smooth curvature into the glassy state. These concerns become especially apparent when examining the residuals plotted in Figure 2, where the residuals of the tanh fit consistently have large deviations near the high-temperature end of the transition. In addition, a nonzero slope can be seen in the glassy state residual, for the bulk film especially, indicating that some of the curvature associated with the lower end of the transition may result in an incorrect α_*G*_ being identified. With that being said, the tanh-based model does provide a continuous function for α(*T*) that describes the main features of the data set with only four fit parameters.

Is there a functional form that can capture the asymmetric features of the α(*T*) data? The tanh function of eq 2 is mathematically equivalent to a standard logistic or sigmoid function that all treat the transition as symmetric:
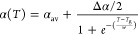
6where  and Δα = α_*M*_ – α_*G*_. In contrast, a *generalized* logistic function can be used to describe a sigmoidal curve without the assumption of symmetry:
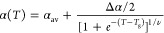
7where the exponent ν plays the role of transition width and asymmetry. A sharper change in α(*T*) at the high-temperature end of the transition is signified by ν > 1, while ν < 1 signifies a sharper change in α(*T*) near the low-temperature asymptote, and ν = 1 is symmetric. For the data shown in Figure 2, the value of ν is much greater than one for both the thick (ν = 9.6) and thin (ν = 17) films. We find that this generalized logistic function does an excellent job of capturing all the features of the glass transition for both thick and thin films with also only four fit parameters.

In Figure 2, we fit the α(*T*) data to the generalized logistic function of eq 7 and show its residual. We find the fit is notably better than the tanh fit, showing much less structure in the glassy state residual, and smaller deviations from the fit around the onset temperature just above *T*_g_. This result is at first glance very encouraging, but comes with a few major caveats. First, the generalized logistic functional form used here does not have a simple analytic integral that could be used as an *h*(*T*) functional form. On top of that, the physical meaning of the parameters are somewhat muddied as a result of the coupling introduced between the exponent ν and the *T*_g_ parameter in the denominator. Specifically, the resulting *T*_g_ value in eq 7 corresponds to a value close to the upper end of the transition, near the *T*_+_ identified by Kawana and Jones, which was found to be film-thickness invariant.^11^ Thus, the *T*_g_ values obtained by fitting eq 7 to the thick and thin film data are within error of each other: 105.4 and 105.6 °C, respectively. While eq 7 represents a more closely fitting model for α(*T*) data, it comes with a few serious limitations that make it unsuitable for modeling *h*(*T*) data and identifying *T*_g_. We will therefore proceed using the tanh model of eq 2 with eq 1 for the *h*(*T*) data during the remainder of our nonlinear fitting analysis.

### 3.4 Bayesian Inference by Hamiltonian Monte Carlo

The main issues with using the Levenberg–Marquardt and other traditional minimization algorithms to solve nonlinear least-squares problems are that the ability to converge to an accurate solution is sensitive to the initial parameter values and their relative scales, and that the performance of the algorithm worsens as the number of independent fit parameters (dimensionality of the chi-squared landscape) increases.^37,47^ A powerful computational approach that has become more accessible in recent years with the development of open-source libraries is Bayesian inference.^40,42^ Bayes’ theorem is applied to efficiently map the chi-squared landscape by guiding a sampling approach like Monte Carlo to identify the regions in parameter space with high probability density (i.e., that are near the chi-squared minimum).^47,48^ Hamiltonian (or Hybrid) Monte Carlo leverages Hamiltonian dynamics to simulate short trajectories in parameter space to improve the selection of the next Monte Carlo move.^41^ These types of methods have recently been employed in various subfields of physics from soft-matter rheology to cosmology for their ability to fit and compare the descriptive power of different complex models for the physical behavior observed.^49,50^

Bayes’ theorem is derived from the definition of conditional probability (that is, the probability of one outcome, given that some set of events has already been observed).^47,48^ It amounts to a statement of invertibility for conditional probabilities: Probability of event *A* conditioned on event *B* multiplied by the probability of event *B* occurring independently is equal to the probability of event *B* conditioned on event *A* multiplied by the probability of event *A* occurring independently:
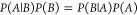
8We are interested in the probability of a set of parameter values Θ = (*T*_g_, *M*, *G*, *w*, *c*), given the experimental *h*(*T*) data ***X*** that have been observed. In terms of our variables Θ and ***X***, Bayes theorem rearranges to
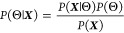
9

The left-hand-side of eq 9, called the *posterior probability*, is what we want to find. It represents the probability distribution of the fit parameters Θ given the experimental data observed ***X***. The conditional probability *P*(***X***|Θ) that appears on the right-hand-side of eq 9 is called the *likelihood function*. It represents the probability distribution for the *h*(*T*) function given a set of parameters Θ. *P*(Θ) is called the *prior distribution*, corresponding to an initial guess of the probability distribution for each of the fit parameters. *Bayesian inference* refers to using Bayes theorem to relate the probability of our initial guess *P*(Θ) to an updated probability *P*(Θ|***X***) conditioned on the data ***X*** having been observed, where with iteration, *P*(Θ|***X***) will converge on the “best fit” (true) probability distribution of the parameter values Θ = (*T*_g_, *M*, *G*, *w*, *c*).

The denominator in eq 9, *P*(***X***), is called the *marginal likelihood* or *model evidence*, which corresponds to the probability of the model itself being the correct representation for the data observed. In Section 3.3, we already addressed that eq 1 may not be the best functional form for *h*(*T*), but represents the best option for an analytical function to fit *h*(*T*) data in practice. Thus, for a given choice of the functional form for *h*(*T*), *P*(***X***) is a constant. In other words, *P*(***X***) amounts to a normalization constant that can be ignored for a given model.

To cast a nonlinear, least-squares fitting problem into a statistical inference problem,^39,51^ the likelihood probability function *P*(***X***|Θ) is defined as a Gaussian distribution of the sum of squared residuals between the experimental data and the model:
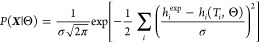
10This is in fact the probability distribution used to define the least-squares fitting procedure based on the central limit theorem,^48^ which simply states that the experimental data *h*_*i*_^exp^ have Gaussian noise that should be within one standard deviation σ of the model *h*_*i*_(*T*_*i*_, Θ) approximately 68% of the time for a given a set of parameters Θ. Note, the likelihood of eq 10 is a multivariate Gaussian distribution with one dimension for each of the five parameters Θ = (*T*_g_, *M*, *G*, *w*, *c*).

The ability within this Bayesian inference framework to specify the initial conditions for the fit parameters as distributions *P*(Θ) for the prior is a significant advantage compared to specifying a single initial value as is done for the standard nonlinear fitting approach. To be as general as possible, we initialize each parameter with a prior distribution that is intended to be “minimally informative” to avoid bias, while allowing parameters to take on any “reasonable” values. We implement this by using a completely uniform distribution for *T*_g_, and broad Gaussian distributions centered at zero for every other variable. It is also useful to put all of the parameters on unity scale for the purposes of optimization and sampling. We can shortcut scaling each of the parameters individually by instead standardizing the data itself, by defining
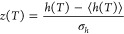
11In so doing, all of the fitting parameters are then on roughly unity scale, allowing us to assign priors that are also on unity scale (i.e., Gaussian distributions with zero mean and unity variance: ).

With these prior initial conditions specified for *P*(Θ), all we need to specify the posterior distribution *P*(Θ|***X***) in eq 9 is the likelihood function of eq 10 with eq 1 as the generative model for *h*_*i*_(*T*_*i*_, Θ) and the experimental data *h*_*i*_^exp^. Then, an algorithm will be used to iteratively sample this posterior distribution *P*(Θ|***X***) in order to map out the converged (“best-fit”) probability distributions of each parameter Θ = (*T*_g_, *M*, *G*, *w*, *c*). This sampling process is accomplished by a Markov Chain Monte Carlo method that optimizes the random selection of each sampled point in parameter space, where we have selected a Hamiltonian Monte Carlo method for this process.^41,51,52^

PyMC is a statistical inference library for Python that is designed for this exact kind of problem.^42^ To set up the problem of finding the posterior distribution, prior distributions are defined for all the variables, then experimental data are provided along with a generative model for that data, which in turn specifies the likelihood function as defined in eq 10. The Bayesian inference is performed by sampling the product of the likelihood and prior. The sampling algorithm chosen is of critical importance here because Metropolis Monte Carlo is prone to random-walk behavior that does not efficiently sample a distribution that has concentrated density in a high-dimensional space.^41^ For these kinds of distributions, a Monte Carlo method that is designed for converging to the regions of high density is highly advantageous. Hamiltonian Monte Carlo (HMC) is one such approach.^41,52^ HMC makes use of storing an ordered list of the parameter values visited (a Markov chain), and uses a short molecular dynamics (MD) simulation (simulating Hamiltonian dynamics with momentum) in parameter space, accelerating down gradients in –log (probability) and decelerating up them, to choose the next Monte Carlo move. This process is repeated for an arbitrary number of steps until the distribution of interest has been characterized in sufficient detail. The result is, instead of a random walk through parameter space as in standard Metropolis Monte Carlo, HMC very efficiently samples regions of high probability density. In general, HMC has two hand-tuning parameters: the size of the time step Δ*t* and the length *L* of the trajectory to run the simulation over (i.e., number of time steps). These parameters must be tuned carefully such that the trajectories are simulated in enough detail to effectively find the regions of high density, but without wasting computational time. We wish to avoid simulating trajectories in finer detail than is necessary compared to the geometry of the distribution being characterized, or running the simulation for longer than is necessary such that the trajectories double back on themselves repeatedly.

One particularly powerful Monte Carlo algorithm built into PyMC is the No-U-Turn Sampler (NUTS).^53^ NUTS is an adaptive implementation of Hamiltonian Monte Carlo (HMC) that eliminates the need to set one or both of the HMC parameters by using a predefined number of iterations to tune the values of *L* and/or Δ*t*. At a conceptual level, the simulation ends and a MC step is made when the trajectory in parameter space simulated both forward and backward in time begins to double back on itself. Precise details can be found in the PyMC documentation, and in the original paper introducing NUTS by Hoffman and Gelman.^42,53^ We use a version of NUTS in our application that adaptively sets both tuning parameters without human intervention. Thus, one key advantage of the approach of casting nonlinear least-squares fitting into a statistical inference problem is that using NUTS requires no hand-tuning.

#### 3.4.1 Bayesian Inference Fitting to Eq 1

**Figure 3:**
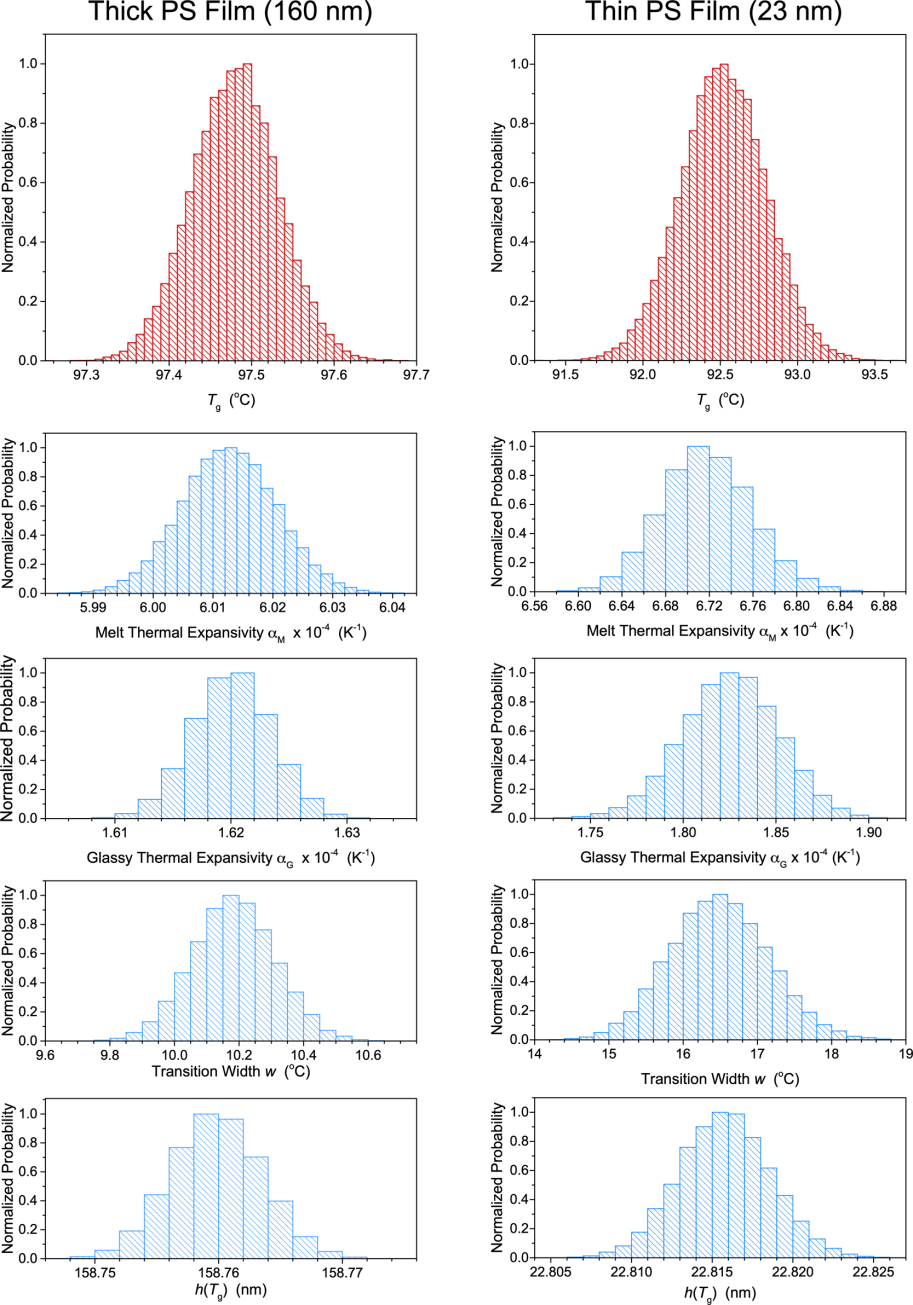
Histograms of the posterior distributions are graphed for the parameter values Θ = (*T*_g_, *M*, *G*, *w*, *c*) obtained from the Bayesian inference fit to eq 1 by sampling over 40,000 Monte Carlo steps. (Bin counts are scaled to the peak count of the distribution.) Left panels are for the bulk PS film (160 nm) and right panels are for the thin PS film (23 nm), where from top to bottom the parameter distributions correspond to *T*_g_, the thermal expansivity of the melt  and glassy  states, the transition width *w*, and the film thickness value at *T*_g_, *h*(*T*_g_). From these distributions, we identify the best fit *T*_g_ = 97.48 ± 0.06 °C for the bulk film and *T*_g_ = 92.5 ± 0.4 °C for the 23 nm thin film.

The Bayesian inference fitting process is run on the *h*(*T*) data of the PS supported films to determine “best-fit” distributions for the parameter values Θ = (*T*_g_, *M*, *G*, *w*, *c*). In Supporting Information we include the Python code to implement this, along with a link to the GitHub repository where the code is applied to the representative data sets we discuss throughout this work. In Figure 3, we graph the posterior distributions of Θ = (*T*_g_, *M*, *G*, *w*, *c*) converged at for bulk and thin film representative data sets for PS films. Histograms obtained after 40,000 MC steps are plotted for each parameter value, determined from four independent Markov chains of 10,000 steps each, where the counts are scaled to the mode of each distribution. We emphasize here that even this relatively large number of MC steps, more than is necessary to obtain distributions that provide sufficient detail, is possible to run in around a minute on a modern workstation PC. The distributions come out to be all Gaussian, as is expected given the mostly independent parameters and the large number of samples. The bulk film, for which the measured film thickness values are more precise relative to the total value, has narrower parameter value distributions compared to the thin film. In addition, we obtain estimates for the thermal expansivities  and  in the melt and glassy states by taking the parameter values for the slopes in *h* versus *T* and dividing by the total film thickness. The standard deviation of the distributions can be used to define an uncertainty for each parameter, with the mean providing a central value. For the bulk film, the mean of the *T*_g_ distribution is located at 97.48 °C with a standard deviation of 0.06 °C, and the thin film *T*_g_ distribution is peaked at 92.5 °C with a standard deviation of 0.4 °C. The mean and standard deviation of *T*_g_ and the other parameter values arrived at are nearly identical to those found by the LM algorithm, however, the Bayesian approach is able to converge to these “best-fit” values with no hand-tuning. We will compare trends in *T*_g_(*h*) with the different fitting methods in detail later on in Section 3.6.

With the Bayesian approach, one also obtains a plethora of statistics about the sampled values and the state of the Markov chains that can be used for diagnosing convergence and model comparison. For example, PyMC comes with the ability to plot “traces” of position in parameter space as a function of iteration number, as well as the difference in energy (potential + kinetic) between these steps in the Hamiltonian MC process. Autocorrelation lag plots can be used to evaluate whether a given parameter has converged, and pair correlation plots can be examined to determine how independent the different parameters are in this chi-squared landscape.

**Figure 4:**
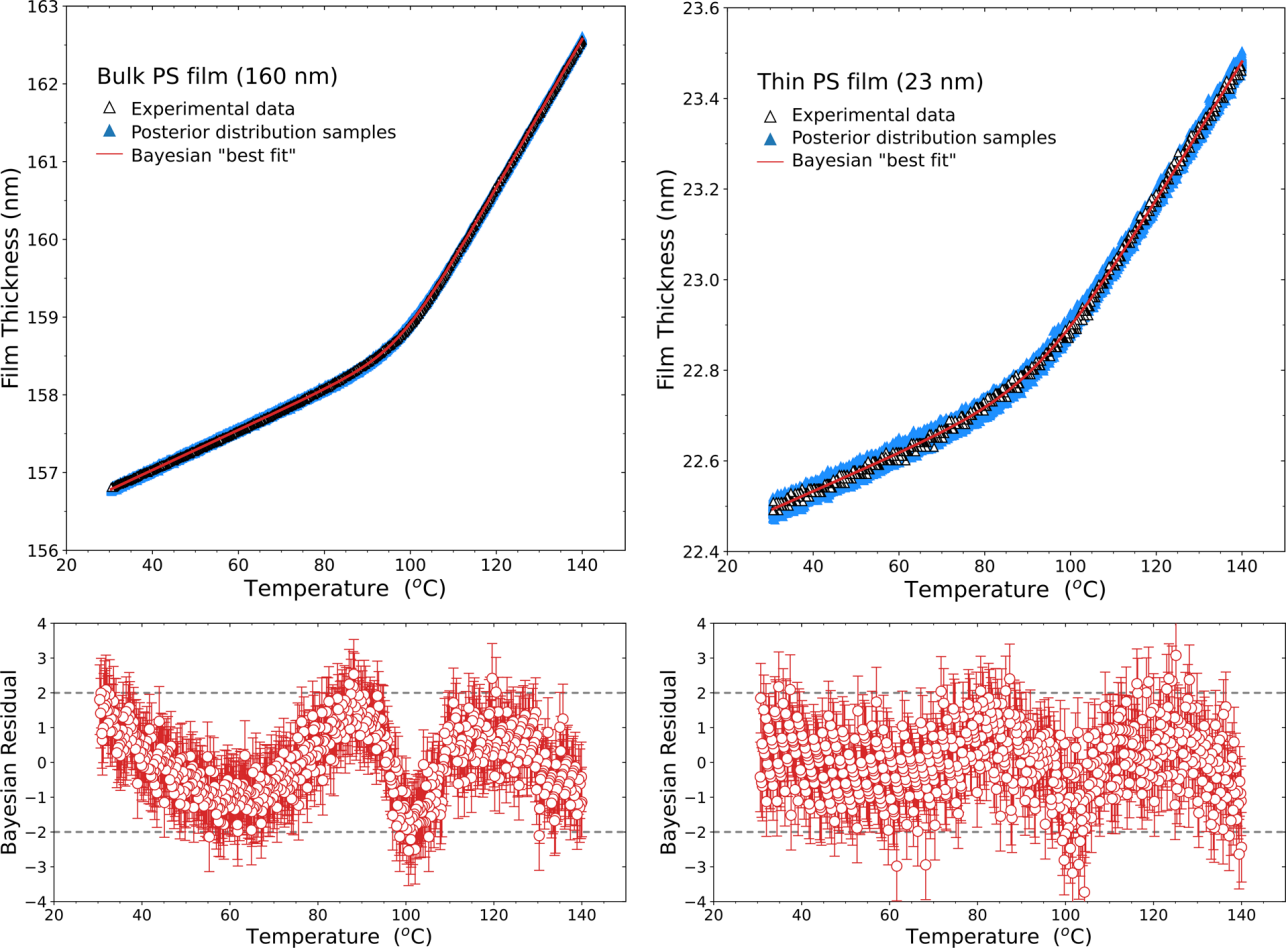
Top panels: Temperature-dependent film thickness *h*(*T*) data for thick (160 nm) and thin (23 nm) PS films with experimental data shown as black open triangles. From the Bayesian inference fit to eq 1, 1000 simulated data sets generated from the converged posterior distributions shown in Figure 3 are plotted using blue solid triangles, with the red curves denoting the “best fit” averaged values. Lower panels are the studentized residuals corresponding to the difference between these “best fit” curves produced by Bayesian inference and the experimental data.

To evaluate the quality of the fits obtained by this Bayesian inference fitting to eq 1, we plot in Figure 4 the experimental film thickness versus temperature data on top of 1000 data sets generated from parameter values drawn from the converged posterior distributions shown in Figure 3. These simulated data are known as “posterior predictive” samples. The solid curves show the mean film thickness versus temperature *h*_*i*_^sim^(*T*_*i*_, Θ) “best fit” curves obtained by averaging the 1000 “posterior predictive” simulated data sets. The spread of the simulated data points seems reasonable and encompasses the experimental data, as one would expect from many iterations of the same measurement. By comparing *h*_*i*_^sim^(*T*_*i*_, Θ) with the experimental *h*(*T*) data, we also plot residuals in Figure 4. Unsurprisingly, these residuals from the Bayesian fitting of eq 1 are nearly identical to the residuals obtained from the LM fitting to eq 1 shown in Figure 1.

The residuals shown in Figure 4 being as similar as they are to those in Figure 1 is not surprising, and demonstrates that when fitting to the same functional form, the Bayesian inference approach recovers the best solution found using the LM algorithm. Because the same eq 1 is used to model *h*(*T*), the quality of the fits, as shown by the residuals, also suffers from the same issues encountered when the fit was obtained using standard LM optimization. The glassy state residual shows significant systematic curvature, and the transition region itself exhibits the same large deviations between the fit and the data as in Figure 1. However, in contrast to the LM approach where we were already at its limits, with the power of the Bayesian inference approach, we are easily able to robustly add additional fit parameters to the *h*(*T*) functional form to account for such systematic deviations.

#### 3.4.2 Bayesian Inference Fitting to the Modified *h*(*T*) Functional Form with Temperature-Dependent Glassy Expansion

To demonstrate the power and flexibility of the Bayesian inference fitting approach, we examine a more complex *h*(*T*) functional form than eq 1 that adds an additional parameter to the glassy state thermal expansion. From Figure 2, we observed that the biggest systematic deviation in the residual was the presence of a nonzero slope to the glassy-state thermal expansion that would suggest we should modify the constant glassy slope *G* in eq 1 to *G*_0_ + *G*_1_ (*T* – *T*_g_). Implementing this change gives us the following equation for *h*(*T*):
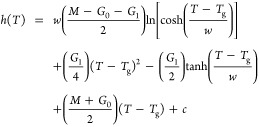
12The other parameters (*M*, *T*_g_, *w*, and *c*) retain their original definition. We note that others in the literature have recently also proposed modifications to eq 1.^54−56^

**Figure 5:**
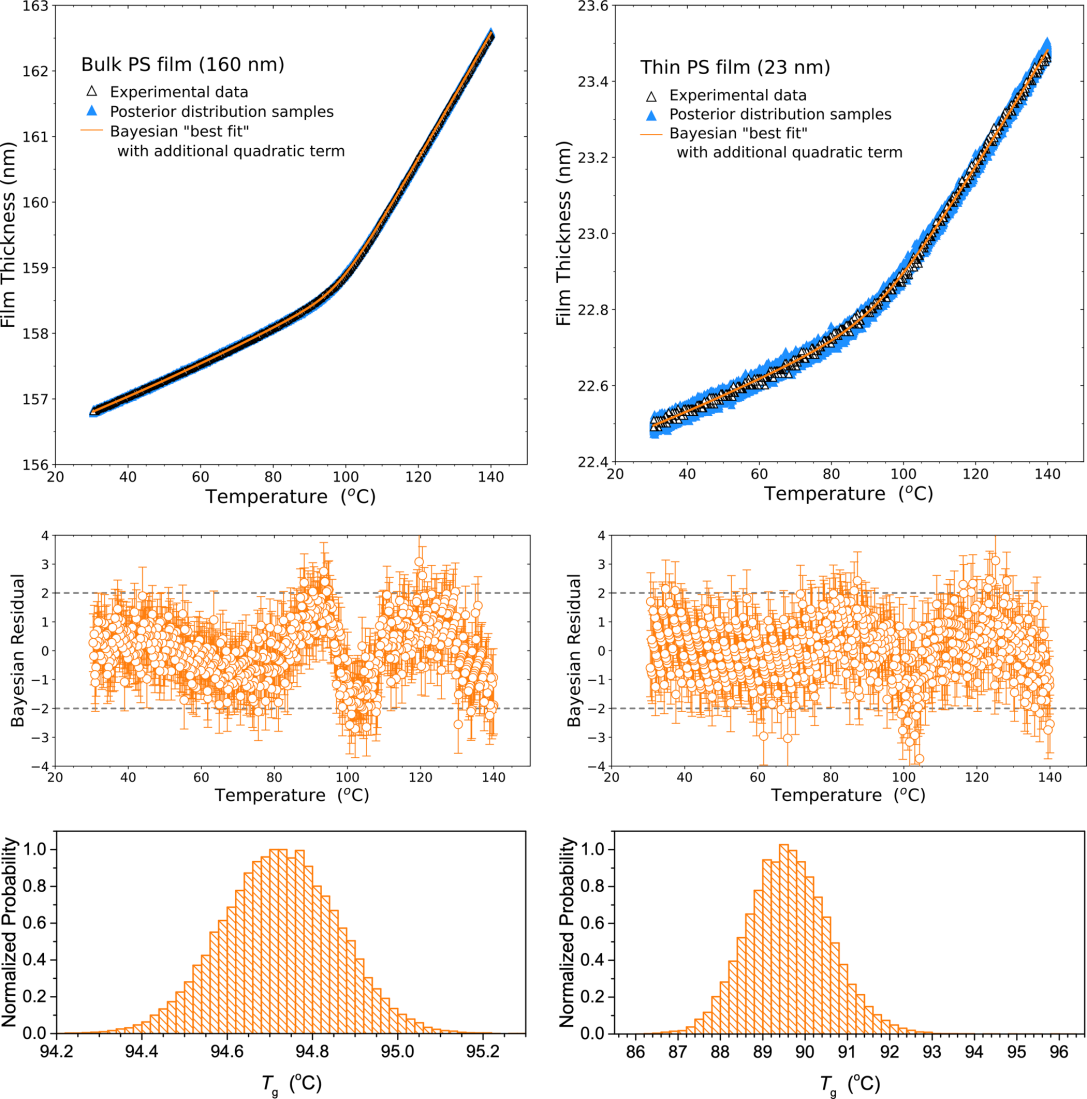
Top panels: Temperature-dependent film thickness *h*(*T*) data for the thick and thin film data fit using Bayesian inference to the modified *h*(*T*) functional form of eq 12 incorporating a temperature-dependent glassy thermal expansion. Experimental data are plotted as black open triangles, with 1000 simulated data sets drawn from the converged posterior distributions plotted as blue solid triangles, and the “best fit” average curve of this Bayesian fit is shown in orange. Middle panels: Studentized residuals of this fit, with the range of −2σ to +2σ marked by gray dashed lines, highlighting that these residuals are well-behaved with the glassy state data for the thick film showing much less systematic deviation from the fit. Bottom panels: Histograms of the converged posterior distributions for *T*_g_, sampled over 40,000 Monte Carlo steps. The best-fit *T*_g_ values are identified as 94.7 ± 0.1 °C for the bulk film and 89.5 ± 1.0 °C for the thin film.

Bayesian inference is able to fit this more elaborate functional form of eq 12 without difficulty with no additional user input, starting from broad (minimally informed) prior distributions. Figure 5 graphs the temperature-dependent film thickness “best fits” and residuals obtained from Bayesian inference fits to eq 12, along with the resulting posterior distributions for *T*_g_. The residuals clearly demonstrate that this model produces a substantially better fit to the glassy state data, especially for the bulk film. The concavity that was plainly visible in the glassy-state residual in Figures 1 and 4 is effectively eliminated in Figure 5. The fit for the thin film does not improve much, as any systematic deviation from simple constant thermal expansion is swamped by the noise in the data. Interestingly, fitting to this augmented eq 12 results in the identification of a somewhat lower *T*_g_ value, where the mean of the *T*_g_ distribution shown for the bulk film is peaked around 94.7 °C, in contrast to the value identified by the fit to eq 1 of 97.5 °C. This difference could be explained by the quadratic term improving the fit to the glassy state portion of the data, and thereby providing greater sensitivity to the low-temperature end of the glassy state far from *T*_g_. The quadratic scaling of *h* with respect to (*T* – *T*_g_) in eq 12 causes the fit to the data near the low-temperature end to be more strongly affected by small changes in the *T*_g_ fitting parameter. Since there is no such effect in the liquid state, still treated as having a constant slope thermal expansion coefficient, this could perhaps contribute to the lower *T*_g_ value.

We note that attempts were made to fit the functional form of eq 12 to the *h*(*T*) data with a conventional LM algorithm. However, only limited success was obtained. Fits could not converge without substantial hand-tuning of initial parameter values and placing constraints on certain parameters, such as the transition width *w*, ultimately resulting in worse χ^2^ values than the simpler fit to eq 1.

Overall, we find the Bayesian framework for nonlinear fitting described here to be robust and versatile. The fitting procedure efficiently handles complex functional forms without needing to guess the correct initial parameter values, while providing meaningful posterior distributions that extract the “best-fit” *T*_g_ value and characterize its uncertainty.

### 3.5 Brute-Force Exhaustive Search of Linear-Fitting Ranges

For comparison, it is worth examining “best-fit” *T*_g_ distributions obtained from the commonly used intersection of linear fits. We employ a brute-force, exhaustive computation to perform multiple iterations of the linear-fit-intersection method by systematically varying the temperature ranges used to fit the glassy and liquid state lines. This characterizes the dependence of *T*_g_ on the choice of fitting windows in a way that provides a more accurate reflection of the true variability in the possible *T*_g_ values obtained when fitting a given data set. Although individual researchers make informed choices of their fitting windows in a reasonable manner, this exercise provides, in the broadest sense, a measure of the possible ranges of *T*_g_ values obtained by many different researchers, which we find ends up encompassing much of the variability in *T*_g_(*h*) trends previously reported in the field.

**Figure 6:**
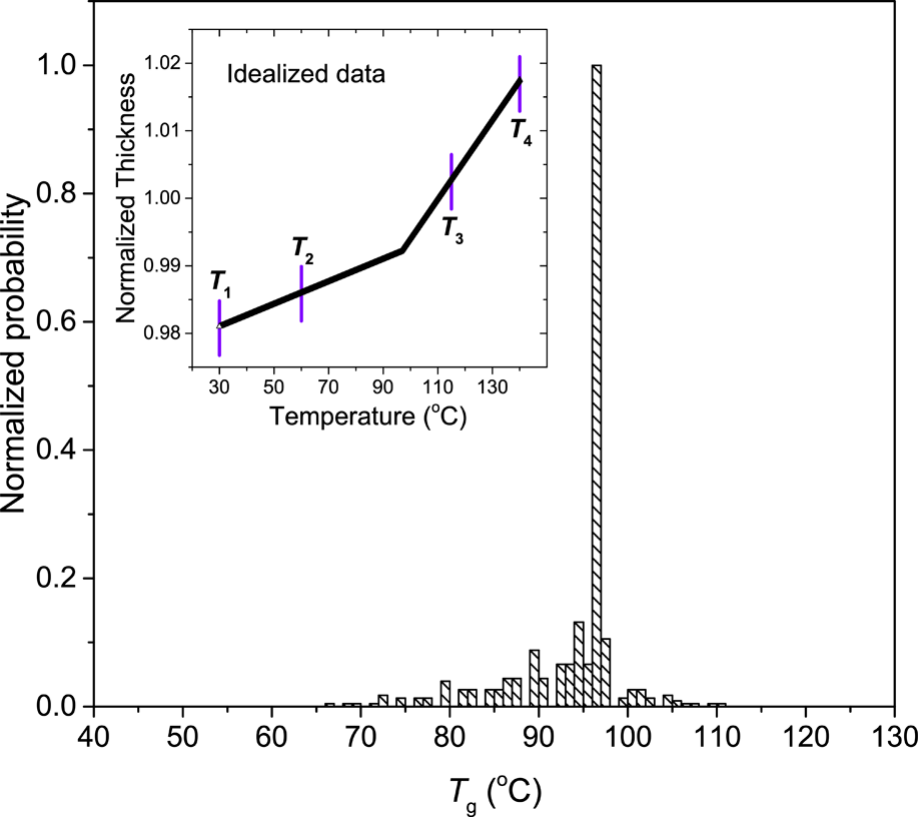
Histogram of *T*_g_ values obtained from the brute-force-search code for an idealized *h*(*T*) data set with no noise, constructed for a bulk film with an instantaneous glass transition at 96.5 °C. The distribution is sharply peaked at the nominal *T*_g_, but other “background” values are still identified. Inset: Idealized film thickness data, with one choice of the fitting window end points *T*_1_, *T*_2_, *T*_3_, *T*_4_ shown.

The fitting windows are enumerated by defining two temperature ranges for the glassy and liquid regimes, as illustrated in the inset of Figure 6. The glassy regime is bounded by *T*_1_ and *T*_2_, and the liquid regime by *T*_3_ and *T*_4_. The code was written to vary these fitting windows in between the lowest temperature *T*_min_ = 30 °C and the highest temperature *T*_max_ = 140 °C of the *h*(*T*) data set, subject to the following conditions:
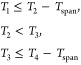
13*T*_span_ corresponds to the minimum size allowed for a possible temperature window, which was set at 5 °C. Both the inner and outer bounds of the temperature windows were individually varied in increments of 10 °C, meaning the available choices for *T*_1_, *T*_2_, *T*_3_, and *T*_4_ were 30, 40, 50, 60, 70, 80, 90, 100, 110, 120, 130, and 140 °C. Linear regressions were then performed for all possible combinations of *T*_1_, *T*_2_, *T*_3_, and *T*_4_ satisfying the conditions listed, and the intersection temperature was computed for each set of linear fits. The *T*_g_ values obtained were then binned into histograms in 1 °C intervals.

For illustration purposes, we start with applying this brute-force computation to an idealized *h*(*T*) data set that represents a bulk thick film with an instantaneous transition between two constant slopes with no measurement noise. The  slopes were chosen to match those for the 160 nm thick representative PS film. Figure 6 displays this idealized data set, along with the computed histogram of *T*_g_ values. This process allows us to evaluate the brute-force code and verify that the central value obtained from the histogram corresponds to the single, known *T*_g_ value of 96.5 °C. Some background counts are present in the histogram of this idealized data set because a small subset of the temperature windows naturally span the transition point, identifying other possible fit values, albeit with much lower frequency. Importantly, this idealized distribution provides a baseline for comparison when evaluating the histograms obtained from real data, where the breadth of the distribution will be impacted by the nonzero width of the glass transition and the noise in the *h*(*T*) data.

**Figure 7:**
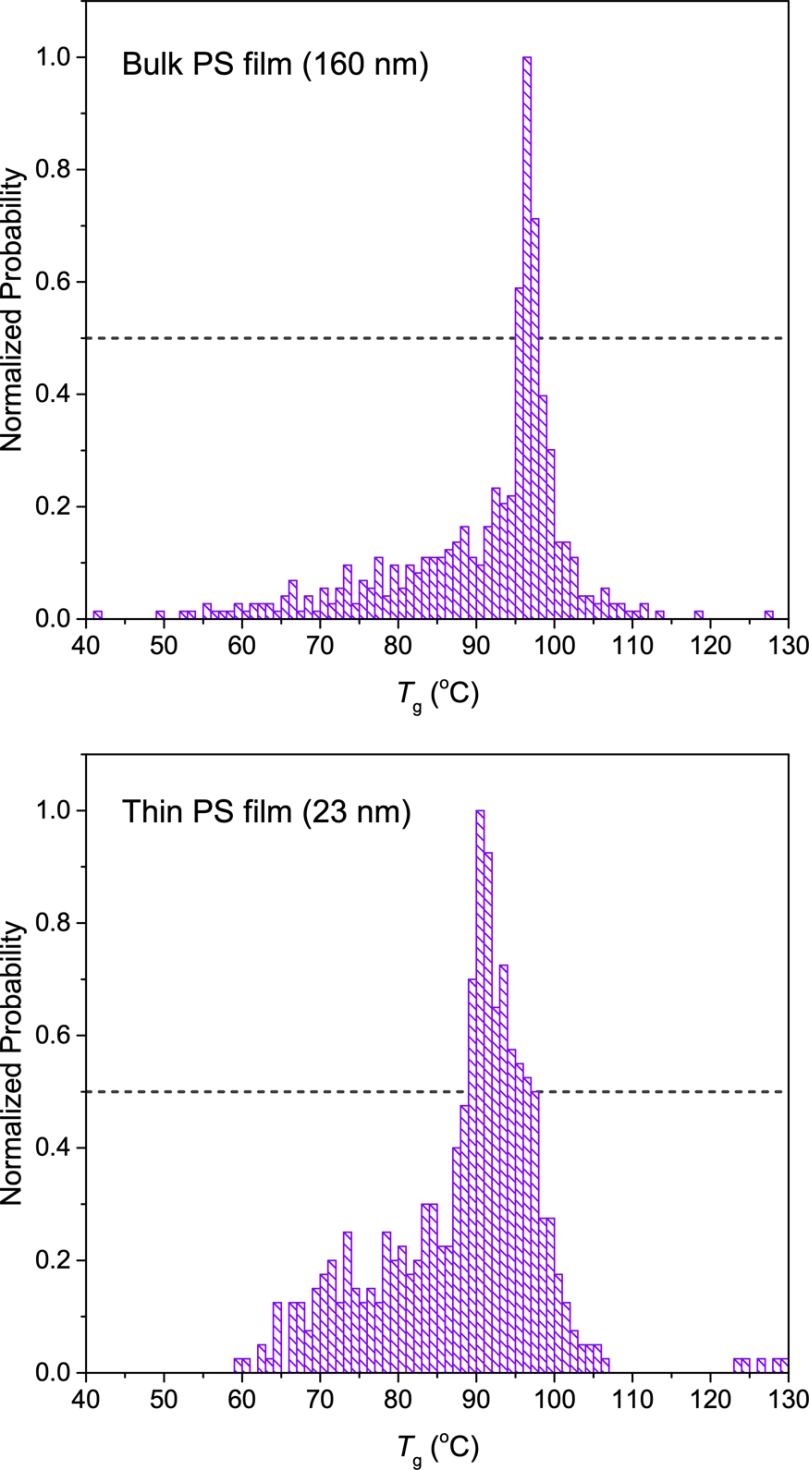
Histograms showing *T*_g_ values determined using a brute-force calculation of many iterations of the linear-fit-intersection method applied to the 160 nm thick and 23 nm thin PS films. The distribution of possible best-fit *T*_g_ values for the thick film is sharply peaked, while the corresponding distribution for the thin film is substantially more spread out, notably toward higher temperatures. Both histograms also show “background” counts that span across the middle of the temperature range, with the large peak associated with *T*_g_ superposed. Dashed gray lines indicate the half-maximum of the distribution from which we choose to define the uncertainty in *T*_g_, where *T*_g_ is obtained from the maximum.

Figure 7 shows the results obtained from the brute-force code applied to the *h*(*T*) data for the representative thick (160 nm) and thin (23 nm) PS films. The histograms of *T*_g_ values, normalized by the mode, both show distinct peaks that one would naturally associate with a “best-fit” *T*_g_. Both distributions also show a wide range of background values arising from how the noise in the *h*(*T*) data and the nonzero transition width impact the identified fit values for the varying selection of temperature windows. These background values appear to be roughly Gaussian, centered at the middle of the temperature range measured. In contrast, a distinctly larger and narrower peak associated with *T*_g_ is superposed on top of these background counts. By comparing the frequency of the background counts present in the histograms from the real and idealized data, we choose a cutoff of the half-maximum of the distribution to identify an uncertainty associated with the central *T*_g_ value. These give us best-fit *T*_g_ values of 96.5 ± 1.5 °C for the bulk 160 nm film and  °C for the 23 nm thin film. The asymmetry of the uncertainty in *T*_g_ is reflected in the histogram, where more bins exceed the half-maximum of the distribution at temperatures higher than the most-probable *T*_g_ value (the mode) than temperatures lower than this value. Reassuringly, the central *T*_g_ values from these distributions agree well with the *T*_g_ values we obtained in Figure 1 from our “most reasonable” selection of fitting windows.

The errors obtained from this brute-force calculation are significantly larger than the fitting error for *T*_g_ obtained using eq 5 when fitting a given *h*(*T*) data set. This demonstrates one of the main flaws of the linear fitting technique: for broad transitions and noisy data, there is more ambiguity in identifying the “best-fit” *T*_g_ value than is represented by a single fit and its associated uncertainty, due to the implicit fitting parameters represented by the end points of the fitting ranges, where the choices of these end points can have an outsize impact on the *T*_g_ value obtained. Thus, the large error determined from this brute-force calculation may better represent the real uncertainty associated with using this linear-fit-intersection method to obtain *T*_g_, particularly in thin films.

### 3.6 Comparing *T*_g_(*h*) for PS Films Obtained from the Different Fitting Methods

In this section, we compare all the different fitting methods we have discussed. Trends in *T*_g_(*h*) and their uncertainties are examined for many different ellipsometric *h*(*T*) data sets for a wide range of PS films. We also discuss the film thickness dependence of the transition width *w*, and the thermal expansion coefficients α_*M*_ and α_*G*_ in the melt and glassy regimes determined from the Bayesian inference fits of eqs 1 and 12. We stress that the uncertainty in *h*(*T*) has been rigorously propagated throughout, and used as weights in each of the fitting methods, thus making an inspection of the film-thickness-dependent trends and uncertainties of these fitting parameters meaningful.

**Figure 8:**
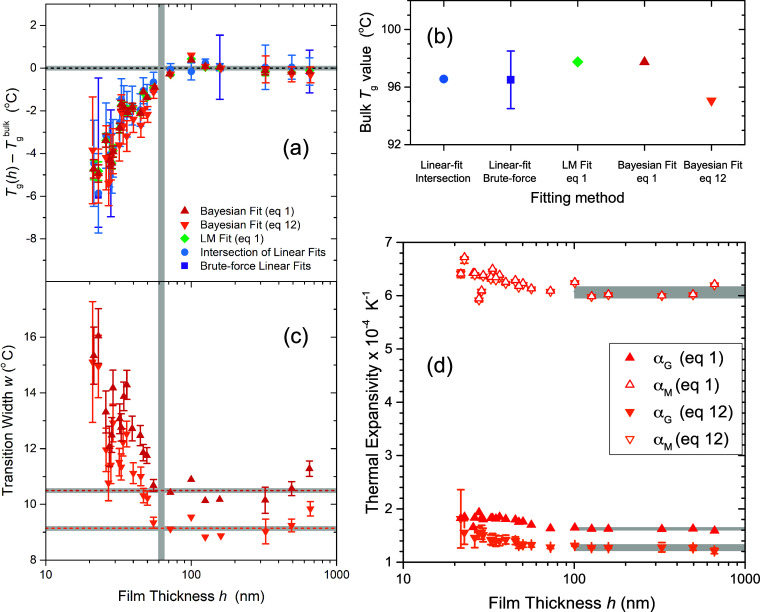
(a) Decrease in *T*_g_ with decreasing film thickness *h*, relative to bulk *T*_g_, for the different fitting methods all showing similar trends in *T*_g_(*h*). Bayesian inference fits to eq 1 (red upward-pointing triangles) and eq 12 (orange downward-pointing triangles) are compared to the standard nonlinear LM fit of eq 1 (green diamonds), the linear-fit intersection method for the user-defined “best” fitting window (blue circles) and a brute-force iteration of many possible fitting windows (purple squares). (b) Bulk *T*_g_ values (average of films with *h* > 100 nm) for the different fitting methods. Error bars are smaller than the symbol sizes, except for the brute-force calculation. (c) Transition width fit parameter *w*(*h*) obtained from Bayesian inference fitting methods is plotted as a function of film thickness, where the horizontal dashed lines indicate the average of the data for *h* > 60 nm for the corresponding set of fits (gray bars highlight uncertainties ±2σ). The vertical gray bar spanning the (a,b) graphs highlights the range of film thickness over which *T*_g_(*h*) and *w*(*h*) begin to deviate from their bulk values. (d) Plot of melt state α_*M*_ (hollow symbols) and glassy state α_*G*_ (solid symbols) thermal expansivities, obtained by the Bayesian inference fits, with horizontal gray bars highlighting bulk values (±2σ) for *h* ≥ 100 nm. The liquid and glassy expansivities are comparatively flat with decreasing film thickness relative to *T*_g_(*h*) and *w*(*h*).

We start in Figure 8a where we compare the deviation of *T*_g_(*h*) from bulk *T*_g_ as reported by each of the fitting methods for PS films with thicknesses ranging from *h* = 21 to 650 nm. Relative to its respective *T*_g_^bulk^, each fitting method exhibits the same trend in *T*_g_(*h*) showing a decrease of ≈5 °C for film thicknesses of *h* ≈ 25 nm. The Bayesian approach to fitting eq 1 provides nearly identical *T*_g_ values and errors to the standard nonlinear LM fit of eq 1. This is unsurprising given that both methods are used to search for the same global minimum in chi-squared parameter space. The linear fit methods identify very similar values to the nonlinear methods, albeit with less precision in general. Notably, the error bars determined from eq 5 for the intersection of the linear fits are larger than those from the nonlinear fits to eq 1. The brute-force method provides an interesting perspective. The *T*_g_ values we identified with manually selected temperature ranges for fitting (such as in Figure 1) correspond closely to the most frequently observed *T*_g_ values in the distributions obtained from the brute-force computation of Figure 7. However, these brute-force distributions suggest wide and sometimes asymmetric errors for the *T*_g_(*h*) data that could be interpreted as a range of plausible *T*_g_ values when accounting for the different fitting windows individual researchers might decide to use. We recognize that the *T*_g_(*h*) shifts shown here are on the smaller end of values reported before in the literature, some of which may be explained by variations in experimental techniques.^12,44^ Although it is also possible that some unintended bias creeps into the manual selection of fitting windows when using the linear-fit intersection method, certainly it appears to us that errors from this fit method are routinely under-reported in the literature. For example, the original Keddie et al. study on thin PS films had no explicit error bars on the *T*_g_ values plotted.^16^ The Bayesian fits to eq 12 provide *T*_g_(*h*) shifts with decreasing film thickness that lie nearly on top of the other fitting methods. The errors on these *T*_g_ values are larger than those for the fits to eq 1, but typically still smaller than the errors associated with the linear fit intersection method. The errors on the *T*_g_ values get particularly large for thin films, consistent with the notion that eq 12 has too many parameters for fitting data from very thin films. This is expected given that any systematic deviations in the residuals for fits to the simpler functional form of eq 1 were already in the noise for the representative thin film data. Where these *T*_g_ fits to eq 12 differ from the other methods is in the absolute value of *T*_g_^bulk^.

Figure 8b compares the bulk *T*_g_ value for each fitting method, determined from an error-weighted average of films with thickness *h* > 100 nm. Both linear fit methods identify *T*_g_^bulk^ as 96.5 °C with an error of ±0.1 °C for the manually selected “most reasonable” fitting windows and ±2.5 °C from the brute-force calculation. The Bayesian and LM fits to eq 1 both found *T*_g_^bulk^ = 97.74 ± 0.02 °C, higher by one degree Celsius. Interestingly, the Bayesian fits to eq 12 identify a notably lower *T*_g_^bulk^ = 95.06 ± 0.06 °C, ≈3 °C lower than that found from fitting to eq 1. This difference is reasonably explained by the functional form of eq 1 not adequately capturing the glassy state behavior near the transition. In particular, it can be seen from the residuals in Figures 1 and 4 that there is systematic concavity in the glassy state when fitting to eq 1, implying that the asymmetry of the transition is being compensated for in the fit by picking a slightly higher glassy state expansion coefficient (as shown in Figure 8d) and transition width. The residuals of the fits to eq 12 in Figure 5 clearly show that this systematic trend is captured by the more complicated functional form in films where the deviation from the fit to eq 1 is large enough relative to the noise of the measurement to be relevant. Due to the quadratic dependence of *h* on *T* – *T*_g_, fitting to eq 12 increases the relative weight of the low-temperature extreme of the data set where *T* – *T*_g_ is largest, resulting in a lower expansivity value being identified, in turn resulting in a lower transition width *w* and thus also a lower *T*_g_.

Figure 8c shows the film thickness dependence of the transition width parameter *w* obtained from Bayesian fits to eqs 1 and 12. To our knowledge the thickness dependence of the transition width for supported PS films has not been explicitly quantified in this manner in the literature previously, although transition width has been used as a fitting parameter^35^ and the span of the transition region has been quantified with *T*^+^ and *T*^–^ values.^11,12^ We find that the transition width is constant for bulk films with *h* > 60 nm near a value of ≈10 °C, which then begins to increase for *h* < 50 nm. The film thickness range over which *T*_g_ and *w* begin to deviate from their bulk values is similar, highlighted by the vertical gray bar. We note interestingly that the *h* = 55 nm data point has reduced *T*_g_ relative to bulk by more than the experimental error, but has a *w* value that is consistent with bulk to within error. The fit transition width *w*(*h*) using eq 1 has the same general thickness dependence as the *w*(*h*) obtained when fitting to eq 12, but shifted upward by about 1 °C. The difference in bulk transition width value between the two models is likely due to the ability to fit the glassy state more accurately with eq 12 compared to eq 1 as evidenced by improved residuals in Figure 5, resulting in lower absolute values of α_*G*_, *w* and *T*_g_.

Figure 8d shows the glassy α_*G*_ and melt α_*M*_ expansivities as a function of film thickness, obtained from the Bayesian fits to eqs 1 and 12. We find that α_*M*_ and α_*G*_ values are comparatively independent of film thickness. The α_*M*_ values are identical between the two models, which makes sense as both equations treat the liquid state as having a single, constant slope. The α_*G*_ values, on the other hand, are notably lower for the fits to eq 12 compared to those of eq 1. The cause of this is that eqs 1 and 12 treat the glassy state differently. Equation 1 treats the glassy state with a constant thermal expansion α_*G*_, as shown in eqs 1 and 2, giving a perfectly linear *h*(*T*) glassy line with a single slope. Equation 12 in contrast treats the glassy thermal expansion as temperature dependent α_*G*_(*T*) = *G*_0_ + *G*_1_ (*T* – *T*_g_), motivated by the curvature in the residual we observed in Figure 4 to the fits of eq 1. When fitting to eq 1, a roughly median value of α_*G*_ is identified in order to minimize the mean-squared error of the fit despite the systematic curvature. Equation 12 can accommodate this curvature, and thus identifies a lower value of α_*G*_ closer to the slope at the low-temperature extreme of the data set. This lower α_*G*_ also results in the slightly lower transition width *w* and *T*_g_ values. For the thick films with *h* ≥ 100 nm, the α_*M*_ and α_*G*_ values obtained using eq 1 are in good agreement with those in the literature. Pye et al. reported α_*G*_ ≈ 1.6 × 10^–4^ K^–1^ and α_*M*_ ≈ 6 × 10^–4^ K^–1^ using ellipsometry for 500 nm thick PS films, which were demonstrated to agree well with handbook volumetric expansivity values when when adjusted to account for constraining effects of the silicon substrate.^8,57^ Compared to the previous analysis done by Kawana and Jones, using a three-line approach to fit numerically differentiated film thickness data from 10 samples, we do not observe the increase in α_*G*_ that they saw with decreasing film thickness,^11^ in accord with other studies in the literature.^12,58^

Overall, Figure 8 shows that the various fitting methods we have evaluated in this study tend to identify deviations from bulk *T*_g_ that are within experimental error of each other, but there is a definite advantage in precision and ability to explicitly fit the transition width *w* provided by the nonlinear fitting methods. The different methods also produce slightly different absolute values of *T*_g_^bulk^, with the eq 12 fits standing out the most from the other approaches. The Bayesian approach is particularly powerful because it is insensitive to the user-provided initial conditions compared to the standard LM algorithm where these initial conditions must be a single point in parameter space close to the global minimum. As a result, Bayesian fitting methods allow more complex models to fit to a given data set, and enable fast batch fitting without respecifying initial conditions across different data sets.

#### 3.6.1 What about Thinner PS Films with *h* < 20 nm?

To properly address thinner films with thicknesses *h* < 20 nm, we need to first revisit the ellipsometer optical layer model fitting that generates the *h*(*T*) data. The quality of the *h*(*T*) data depends on the ability of the optical layer model to accurately and unambiguously fit the ellipsometer’s raw Ψ(λ) and Δ(λ) data to determine the film thickness *h* and refractive index *n*(λ) as described in eq 3. As the films become thinner, the wavelength dependence of Ψ(λ) and Δ(λ) become more featureless resulting in more ambiguous fits and ultimately noisier *h*(*T*) data.^20^ For example, the RMS fitting error in *h* provided by the Woollam software, δ*h*, is 0.013 nm for 10 nm thick films, compared with 0.013 nm for the 23 nm thick and 0.009 nm for the bulk 160 nm representative films. Even though the δ*h* error in *h* is the same for the 10 and 23 nm thick films, the effective noise in the data δ*h*/*h* will be twice as large for the 10 nm films because the thickness is half as large. In addition, the polarization change defining Ψ(λ) and Δ(λ) becomes less dependent on the material’s dispersion, i.e., the wavelength dependence of the refractive index *n*(λ), for thinner films because the light spends so little time passing through the film such that the polarization change comes primarily from the reflection at the interfaces.^20,59^ To address this, it is common for researchers to hold the Cauchy *B* parameter fixed, along with the *C* parameter, in eq 3 for the *n*(λ) parametrization. We have done this for films with thickness *h* ≤ 10 nm, holding *B* = 0.00745 and *C* = 0.00038 at the bulk values,^20^ finding it has very little impact on the temperature-dependent *h*(*T*) data for these ultrathin films as one would expect. It is worth noting that also holding *A* constant would completely remove the temperature dependence from the refractive index and that does alter the behavior of the *h*(*T*) data.

**Figure 9:**
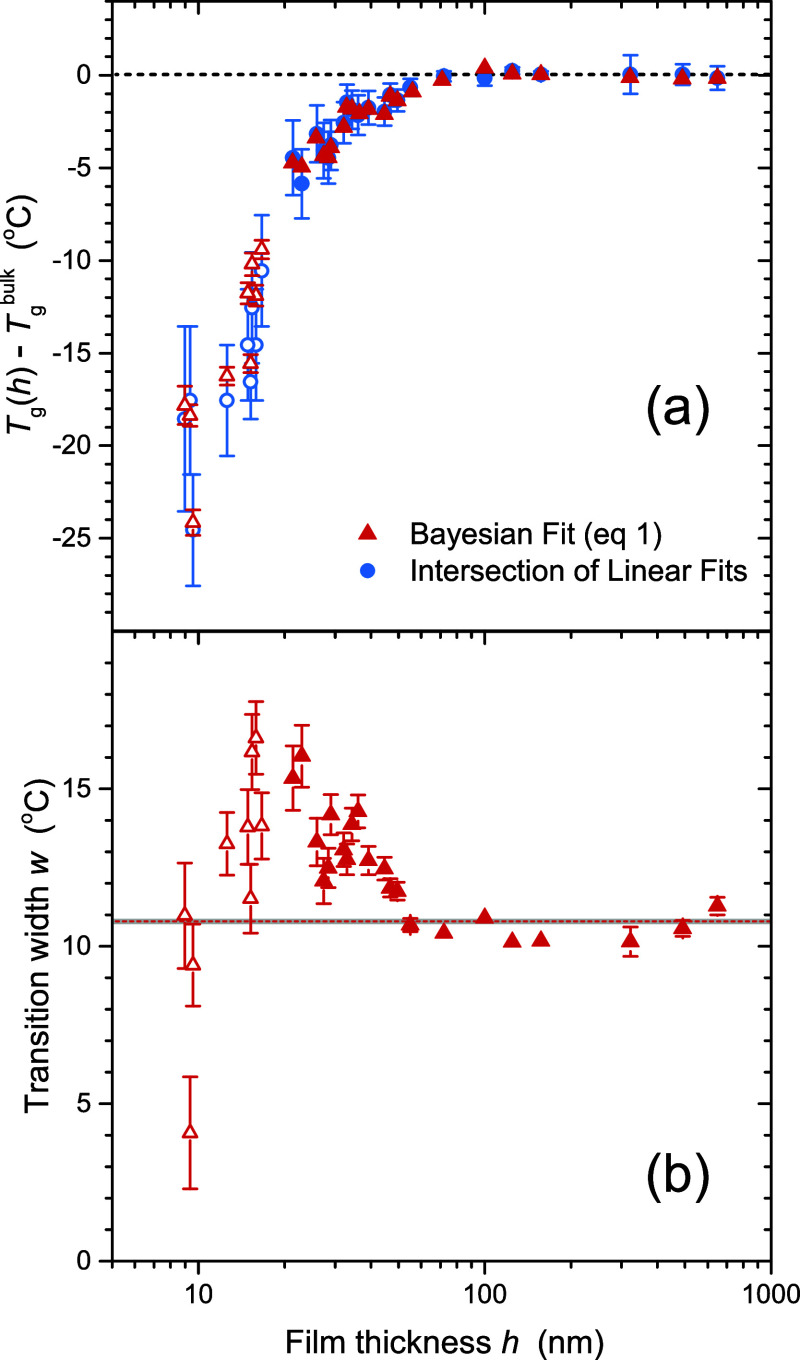
(a) Plot of *T*_g_(*h*) for PS thin films extended down to thicknesses *h* < 20 nm (open symbols), while the solid symbols correspond to the thicker film data shown in Figure 8. The Bayesian fits to eq 1 continue to provide reliable fits with small error bars for *T*_g_, while the common linear-fit intersection method obtained by user-defined “best” fitting windows struggles showing an increasing error in *T*_g_ for thinner films. (b) The transition width *w*(*h*) obtained from the Bayesian fits surprisingly shows a down turn in thin films *h* < 20 nm, which likely indicates a narrowing of the distribution of relaxation times in thin films.

In Figure 9 we plot *T*_g_(*h*) and *w*(*h*) for PS thin films where we have extended the data set to include film thicknesses *h* < 20 nm. *T*_g_(*h*) shows the large reduction in *T*_g_ from bulk as film thickness is reduced that has been well characterized in the literature. The Bayesian fits to eq 1 are able to fit the *h*(*T*) data for these thin films with ease, retaining the small error in *T*_g_. In contrast, the commonly employed linear-fit intersection method, using the “best” user-defined fitting windows, struggles with the thinner films showing increasingly larger error bars in *T*_g_ with decreasing film thickness. Both fitting methods find that very thin films with *h* ≈ 9 nm exhibit larger sample-to-sample variability in *T*_g_(*h*). Interestingly, the trend in the transition width *w*(*h*) that is reliably obtained from the Bayesian fits shows an unexpected trend below *h* = 20 nm that strongly indicate a persistent decrease in the transition width. This probably reflects a reduction in the distribution of relaxation times in these very thin films, a finding that would be consistent with the previous observation by Ellison and Torkelson that very thin PS films have a less reduced local *T*_g_ at the free surface and appear to be more dynamically homogeneous.^60^

### 3.7 Bayesian Inference Fitting Applied to P2VP Thin Films

**Figure 10:**
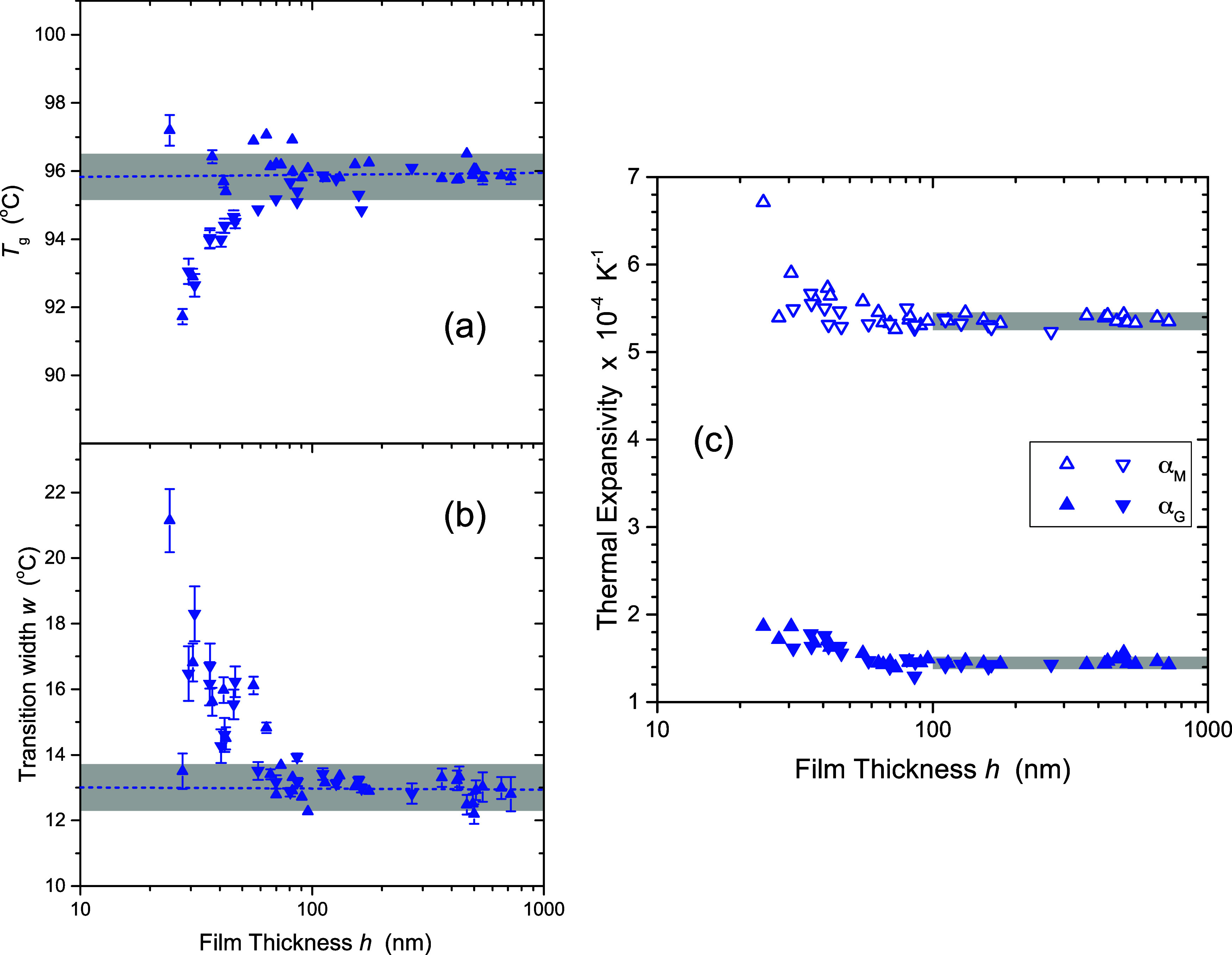
Bayesian inference fitting approach using eq 1 applied to P2VP thin films supported on silicon. Values of (a) *T*_g_, (b) transition width *w*, and (c) thermal expansivity values for the α_*M*_ melt state (hollow symbols) and α_*G*_ glassy state (solid symbols) are plotted as as a function of film thickness *h*. Data for two identical molecular weights are shown as upward (*M*_w_ = 650 kg/mol) and downward (*M*_w_ = 643 kg/mol) pointing triangles. Horizontal dashed lines indicate the average of the data for *h* > 100 nm for the corresponding set of fits, gray bars highlight uncertainties of ±2σ. *T*_g_(*h*) and *w*(*h*) begin to deviate from bulk around the same critical thickness of ≈60 nm as that for PS, but show significant sample-to-sample variability for thin films. For P2VP, the breadth of the transition in thin films *w* > 15 °C, likely associated with the strong gradient in dynamics, far exceeds the ≈5 °C variability in *T*_g_(*h*).

Having benchmarked our Bayesian inference fitting approach on PS thin films, we now apply this to a different system, a set of data from poly(2-vinylpyridine) (P2VP) thin films to assess changes in *T*_g_(*h*), *w*(*h*), and thermal expansivities α_*M*_ and α_*G*_ with decreasing film thickness. Figure 10 shows values of *T*_g_(*h*) for P2VP films obtained from Bayesian fits to eq 1. Unlike the PS data that clearly show a reduction in *T*_g_(*h*) with decreasing film thickness, P2VP films show significant variability in *T*_g_(*h*) from sample-to-sample in the thin film regime (*h* < 80 nm). Bulk films with thicknesses *h* > 100 nm give an average bulk *T*_g_ of 95.8 ± 0.3 °C and transition width of 13.0 ± 0.4 °C. Although *T*_g_(*h*) does not change substantially with decreasing film thickness, the transition width *w*(*h*) increases sharply as the film thickness is reduced. As shown in Figure 10b, *w*(*h*) increases from its bulk value of 13.0 to 21 °C, a much larger change than that seen for PS. This likely reflects the very large gradient in dynamics occurring in these films.

The width *w* of the glass transition is expected to broaden significantly for P2VP films in the regime of film thicknesses for which we have data, as has been previously shown by Glor et al.^29^ Their study even suggested that for ultrathin films (*h* < 20 nm), the transition may in fact broaden to the point that two distinct *T*_g_s appear. Glor et al. characterized the breadth of the transition by identifying *T*_+_ and *T*_–_ values, corresponding to the start and end of the transition on cooling, finding transition breadths (*T*_+_ – *T*_–_) of up to 51 K for 16 nm thick films.^29^ Their definition of transition breadth would roughly correspond to 2*w* from our fits, in reasonable agreement. Whether interpreted as two separate *T*_g_ values, or as a single very broad transition, this broadness speaks to the strong difference in local dynamics near the free surface and substrate interfaces. Because we are implementing the Bayesian inference fitting using eq 1 that only allows for a single transition, it is possible this transition breadth may impact the Bayesian fitting routine’s ability to identify a single *T*_g_ value, and thus contribute to the variability in the *T*_g_ values identified for thin films.

Figure 10c graphs the expansivities for the melt α_*M*_ and glassy α_*G*_ states obtained from the Bayesian fits to the P2VP data. The trends in α_*M*_ and α_*G*_ with decreasing film thickness are overall quite similar to those observed for PS films. The higher variability in *T*_g_ and *w* parameters means there is somewhat more spread observed in the α_*M*_ and α_*G*_ values in the thin film regime compared to PS. The bulk values we obtain for P2VP are α_*M*_ = (5.36 ± 0.05) × 10^–4^ K^–1^ and α_*G*_ = (1.45 ± 0.04) × 10^–4^ K^–1^. Both values are slightly lower than those obtained for PS, and appear to agree reasonably well with the plateau values shown by Glor et al.^29^

The behavior of how *T*_g_(*h*) changes in thin P2VP films with decreasing thickness is considerably less well established in the literature than that for PS thin films. A series of older papers reported large increases in *T*_g_(*h*) with decreasing thickness,^61−63^ while newer studies report small *T*_g_(*h*) decreases coupled with strong broadening of the transition in thin films.^29,64^ Unfortunately what conclusions can be drawn from these studies are limited, as in some cases the data reported are for only 4–5 film thicknesses. In the present work, we plot *T*_g_(*h*) values fit from 49 different samples, where we find a high degree of sample-to-sample variability, even though the fitting error in *T*_g_ for any given *h*(*T*) data set is small (around ±0.2–0.4 °C). The underlying cause of this variability is unclear, but may reflect such a broadened transition that a single *T*_g_ value is no longer meaningful. The breadth of the transition in these thin films (*w* > 15 °C) far exceeds the variability in *T*_g_(*h*) values (≈5 °C). If we examine a group of *h*(*T*) data at film thicknesses of ≈28 nm that exhibits the highest variability, we find noticeable differences between the *h*(*T*) data themselves that suggest drawing conclusions from a single data set may be unreliable. For example, it is possible with fewer data points that our results might convincingly suggest a consistent *T*_g_(*h*) trend that increased or decreased. However, by comparing data from a large number of different films it becomes clear that sample-to-sample variability is high. The strongly competing free surface and substrate interactions in thin P2VP films appear to result in such a strong dynamical gradient that the resulting *T*_g_ value identified from a given measurement in thin films is highly variable.^29,64−68^ In such cases, multiple repeated measurements, aided by an unbiased automated fitting of *T*_g_, provide a more accurate sampling of the glass transition in thin films.

## 4 Conclusions

A Bayesian inference fitting method using Hamiltonian Monte Carlo was developed leveraging open-source Python resources that eliminates the key drawbacks of standard Levenberg–Marquardt nonlinear fitting. This new Bayesian fitting method to determine *T*_g_ was applied to a set of film thickness *h*(*T*) data collected by ellipsometry for supported PS and P2VP films spanning a wide range of thicknesses previously published by our group.^20,45^ Formulating least-squares fitting as a Bayesian inference problem involves defining the likelihood probability function as a Gaussian distribution of the sum of squared residuals between the experimental data and a functional form that describes the *h*(*T*) data. Hamiltonian Monte Carlo is then used to efficiently map the regions of high probability density in this effective chi-squared landscape. This robust global optimization from unbiased initial starting conditions provides “best-fit” probability distributions for each fit parameter.

An existing functional form by Dalnoki-Veress et al.,^18^eq 1, was used as a starting point to fit the *h*(*T*) data to obtain trends in *T*_g_, transition width *w*, and thermal expansivity α_*M*_ and α_*G*_ values as a function of film thickness. The Bayesian fitting provides meaningful values for these parameters and their uncertainties allowing us to juxtapose the trends in *T*_g_(*h*) and *w*(*h*) for PS films. The thickness dependence of the transition width *w*(*h*) increases for thin films, indicating the glass transition is broadened, deviating from bulk around the same critical thickness of ≈50–60 nm as *T*_g_(*h*) itself. However, for ultrathin films with *h* < 20 nm, the transition width *w*(*h*) was interestingly found to decrease again suggesting a narrowing of the dynamical gradient, as one previous report has suggested.^60^ In contrast, we find the thermal expansion coefficients to be comparatively flat with respect to film thickness, in agreement with some of the literature^12,58^ and with bulk values matching existing literature and handbook values.^8,57^ When applied to P2VP thin films, the Bayesian fitting approach identifies a larger broadening of the glass transition with decreasing film thickness as compared to PS, consistent with existing reports in the literature.^29^ The values of *T*_g_(*h*) appear to decrease slightly with decreasing film thickness, in good agreement with some recent measurements.^29,64^

The accuracy of eq 1 to describe *h*(*T*) data for thin PS films was examined by numerically differentiating film thickness data and comparing to different models for . We find that the standard tanh functional form that underpins eq 1 is the best among models for α(*T*) that have an analytic integral form that can be used to fit *h*(*T*). However, we also leveraged our Bayesian fitting approach to investigate the use of a quadratic term in the glassy-state film thickness temperature dependence of eq 12. For sufficiently precise data such as that obtained for films with thicknesses down to about 100 nm, we found that a quadratic term substantially improves the quality of fits and does not affect the ability of the Bayesian inference method to converge to a solution. In contrast, the standard nonlinear fitting (LM) approach is unable to handle the additional parameter for many typical ellipsometry data sets, leading to worse χ^2^ values even when other parameters are held constant or constrained.

These benefits of Bayesian inference fitting are demonstrated by comparing to existing fitting methods to obtain *T*_g_ from temperature-dependent film thickness *h*(*T*) data commonly used in the polymer science literature. Perhaps unsurprisingly, a standard LM nonlinear fitting approach to eq 1 is able to reproduce the same fits as the Bayesian inference fitting method, however effort is needed to identify correct initial guesses for the parameter values to obtain the correct global minimum. Notably, we find that fitting to eq 1 is vastly superior to the frequently used approach of identifying *T*_g_ via the intersection of two linear fits to the liquid and glassy regimes because fitting windows need to be manually selected. In contrast, eq 1 allows all the available data to be fit, including through the transition itself, where the transition width can be explicitly modeled without the requirement of identifying fitting windows that exclude the data around the transition. We further evaluated this common linear-fit-intersection method by implementing a brute-force iterative approach to select fitting windows that created an effective distribution of *T*_g_ values that demonstrate the sensitivity of *T*_g_ to fitting window boundaries. Fortunately, the *T*_g_(*h*) values we identified by manually selecting “most reasonable” fitting windows were found to correspond closely to the most frequently observed *T*_g_ value, i.e. the peak in the *T*_g_ distribution, obtained by the brute-force approach. However, the distributions of *T*_g_ values obtained by this brute-force approach demonstrate that there is a substantial amount of uncertainty in *T*_g_ that exists because the end points of the linear fitting windows can be varied, and this uncertainty is not possible to quantify with only a single pair of fit lines. We would recommend that if the linear-fit-intersection method is used, several different fitting windows should be explored to ensure that the obtained *T*_g_ is robust and to accurately estimate the uncertainty. This would in effect provide a systematic sensitivity analysis to this fitting approach. From Figure 8 we can conclude that a robustly obtained *T*_g_ from the linear-fit-intersection method agrees reasonably well with the nonlinear fit methods, although with larger uncertainty.

Ideally, to accurately quantify *T*_g_(*h*) trends from *h*(*T*) data for thin polymer films, errors are rigorously propagated through each fitting operation to obtain physically meaningful uncertainties on the fit parameters themselves, and to enable the quality of the fits to be evaluated through residual analysis. The Bayesian inference approach makes this a more practical, straightforward task requiring much less human supervision and input than existing methods for fitting *T*_g_. Overall, the Bayesian approach to fitting trades a small increase in conceptual and computational complexity, compared to standard nonlinear approaches, for a litany of benefits: substantially improved convergence, the ability to specify a distribution of parameter values as an initial condition rather than a single point, a smarter sampling algorithm (NUTS) to map the probability distributions of the parameters, and the ability to fit to more complex models while providing assurance that the solution identified corresponds to the global minimum in chi-squared. Due to its insensitivity to the supplied initial conditions, it is also relatively easy to automate the Bayesian fitting process to batch-fit many data sets at once. The ability to automate data fitting in a reliable and robust manner, especially on data sets that are noisy or challenging to fit, is particularly important for machine learning implementation of polymer materials characterization and discovery.^13−15^ In Supporting Information, we link to a Github repository PyTg that provides the Bayesian inference fitting code we developed in Python using PyMC, an open-source library for statistical inference.^42^ This code can be easily adapted to implement the Bayesian fitting method to any number of different functional forms to fit other experimental data.

## Supporting Information Available

Github repository PyTg’s readmefile and Python code for the Bayesian inference fitting using Hamiltonian Monte Carlo. Link to Github repository PyTg: https://github.com/jmerri8/PyTg (PDF)

## References

[ref1] Weber M. F.; Stover C. A.; Gilbert L. R.; Nevitt T. J.; Ouderkirk A. J. Giant Birefringent Optics in Multilayer Polymer Mirrors. Science 2000, 287, 2451–2456. 10.1126/science.287.5462.2451. 10741958

[ref2] Léger L.; Creton C. Adhesion mechanisms at soft polymer interfaces. Philosophical Transactions of the Royal Society A: Mathematical, Physical and Engineering Sciences 2008, 366, 1425–1442. 10.1098/rsta.2007.2166. 18156130

[ref3] Mundra M. K.; Donthu S. K.; Dravid V. P.; Torkelson J. M. Effect of Spatial Confinement on the Glass-Transition Temperature of Patterned Polymer Nanostructures. Nano Lett. 2007, 7, 713–718. 10.1021/nl062894c. 17288488

[ref4] Xie K.; Fu Q.; Qiao G. G.; Webley P. A. Recent progress on fabrication methods of polymeric thin film gas separation membranes for CO2 capture. J. Membr. Sci. 2019, 572, 38–60. 10.1016/j.memsci.2018.10.049.

[ref5] McKenna G. B. In Comprehensive Polymer Science and Supplements, Vol. 2: Polymer Properties; Booth C.; Price C., Eds.; Pergamon Press, 1989; pp 311–362.

[ref6] Filliben J. L.; McKinney J. E. Confidence Limits for the Abscissa of Intersection of Two Linear Regressions. Journal of Research of the National Bureau of Standards B: Mathematical Sciences 1972, 76B, 179–192. 10.6028/jres.076B.013.

[ref7] Baker E. A.; Rittigstein P.; Torkelson J. M.; Roth C. B. Streamlined ellipsometry procedure for characterizing physical aging rates of thin polymer films. J. Polym. Sci., Part B: Polym. Phys. 2009, 47, 2509–2519. 10.1002/polb.21861.

[ref8] Pye J. E.; Roth C. B. Physical Aging of Polymer Films Quenched and Measured Free-Standing via Ellipsometry: Controlling Stress Imparted by Thermal Expansion Mismatch between Film and Support. Macromolecules 2013, 46, 9455–9463. 10.1021/ma401872u.

[ref9] White R. P.; Buculei D.; Beale A. M. J. M.; Goovaerts I.; Keddie J. L.; Lipson J. E. G. Spectroscopic ellipsometry as a route to thermodynamic characterization. Soft Matter 2022, 18, 6660–6673. 10.1039/D2SM00959E. 36004577

[ref10] Forrest J. A.; Dalnoki-Veress K. The glass transition in thin polymer films. Adv. Colloid Interface Sci. 2001, 94, 167–195. 10.1016/S0001-8686(01)00060-4.

[ref11] Kawana S.; Jones R. A. L. Character of the glass transition in thin supported polymer films. Phys. Rev. E 2001, 63, 021501 10.1103/PhysRevE.63.021501. 11308492

[ref12] Kim S.; Hewlett S. A.; Roth C. B.; Torkelson J. M. Confinement effects on glass transition temperature, transition breadth, and expansivity: Comparison of ellipsometry and fluorescence measurements on polystyrene films. Eur. Phys. J. E 2009, 30, 83 10.1140/epje/i2009-10510-y. 19784679

[ref13] Beaucage P. A.; Sutherland D. R.; Martin T. B. Automation and Machine Learning for Accelerated Polymer Characterization and Development: Past, Potential, and a Path Forward. Macromolecules 2024, 57, 8661–8670. 10.1021/acs.macromol.4c01410.

[ref14] Jha A.; Chandrasekaran A.; Kim C.; Ramprasad R. Impact of dataset uncertainties on machine learning model predictions: the example of polymer glass transition temperatures. Modell. Simul. Mater. Sci. Eng. 2019, 27, 024002 10.1088/1361-651X/aaf8ca.

[ref15] Tao L.; Varshney V.; Li Y. Benchmarking Machine Learning Models for Polymer Informatics: An Example of Glass Transition Temperature. J. Chem. Inf. Model. 2021, 61, 5395–5413. 10.1021/acs.jcim.1c01031. 34662106

[ref16] Keddie J. L.; Jones R. A. L.; Cory R. A. Size-Dependent Depression of the Glass Transition Temperature in Polymer Films. Europhysics Letters (EPL) 1994, 27, 59–64. 10.1209/0295-5075/27/1/011.

[ref17] Keddie J. L.; Jones R. A. L.; Cory R. A. Interface and surface effects on the glass-transition temperature in thin polymer films. Faraday Discuss. 1994, 98, 219 10.1039/fd9949800219.

[ref18] Dalnoki-Veress K.; Forrest J. A.; Murray C.; Gigault C.; Dutcher J. R. Molecular weight dependence of reductions in the glass transition temperature of thin, freely standing polymer films. Phys. Rev. E 2001, 63, 031801 10.1103/PhysRevE.63.031801. 11308668

[ref19] Pye J. E.; Roth C. B. Two simultaneous mechanisms causing glass transition temperature reductions in high molecular weight freestanding polymer films as measured by transmission ellipsometry. Phys. Rev. Lett. 2011, 107, 235701 10.1103/PhysRevLett.107.235701. 22182101

[ref20] Huang X.; Roth C. B. Changes in the temperature-dependent specific volume of supported polystyrene films with film thickness. J. Chem. Phys. 2016, 144, 234903 10.1063/1.4953855. 27334190

[ref21] Tsui O. K. C.; Russell T. P.; Hawker C. J. Effect of Interfacial Interactions on the Glass Transition of Polymer Thin Films. Macromolecules 2001, 34, 5535–5539. 10.1021/ma000028v.

[ref22] Ellison C. J.; Mundra M. K.; Torkelson J. M. Impacts of Polystyrene Molecular Weight and Modification to the Repeat Unit Structure on the Glass Transition-Nanoconfinement Effect and the Cooperativity Length Scale. Macromolecules 2005, 38, 1767–1778. 10.1021/ma047846y.

[ref23] Rauscher P. M.; Pye J. E.; Baglay R. R.; Roth C. B. Effect of Adjacent Rubbery Layers on the Physical Aging of Glassy Polymers. Macromolecules 2013, 46, 9806–9817. 10.1021/ma401498m.

[ref24] Merrill J. H.; Li R.; Roth C. B. End-Tethered Chains Increase the Local Glass Transition Temperature of Matrix Chains by 45 K Next to Solid Substrates Independent of Chain Length. ACS Macro Lett. 2023, 12, 1–7. 10.1021/acsmacrolett.2c00582. 36516977

[ref25] Rittigstein P.; Torkelson J. M. Polymer–nanoparticle interfacial interactions in polymer nanocomposites: Confinement effects on glass transition temperature and suppression of physical aging. J. Polym. Sci., Part B: Polym. Phys. 2006, 44, 2935–2943. 10.1002/polb.20925.

[ref26] Baglay R. R.; Roth C. B. Communication: Experimentally determined profile of local glass transition temperature across a glassy-rubbery polymer interface with a Tg difference of 80 K. J. Chem. Phys. 2015, 143, 111101 10.1063/1.4931403. 26395676

[ref27] Lipson J. E. G.; Milner S. T. Local and Average Glass Transitions in Polymer Thin Films. Macromolecules 2010, 43, 9874–9880. 10.1021/ma101099n.

[ref28] Napolitano S.; Glynos E.; Tito N. B. Glass transition of polymers in bulk, confined geometries, and near interfaces. Rep. Prog. Phys. 2017, 80, 036602 10.1088/1361-6633/aa5284. 28134134

[ref29] Glor E. C.; Angrand G. V.; Fakhraai Z. Exploring the broadening and the existence of two glass transitions due to competing interfacial effects in thin, supported polymer films. J. Chem. Phys. 2017, 146, 203330 10.1063/1.4979944. 28571332

[ref30] Roth C. B.; Dutcher J. R. Glass transition temperature of freely-standing films of atactic poly(methyl methacrylate). Eur. Phys. J. E 2003, 12, 103–107. 10.1140/epjed/e2003-01-024-2. 15011026

[ref31] Roth C. B.; Pound A.; Kamp S. W.; Murray C. A.; Dutcher J. R. Molecular-weight dependence of the glass transition temperature of freely-standing poly(methyl methacrylate) films. Eur. Phys. J. E 2006, 20, 441–448. 10.1140/epje/i2006-10034-0. 16957829

[ref32] Raegen A. N.; Massa M. V.; Forrest J. A.; Dalnoki-Veress K. Effect of atmosphere on reductions in the glass transition of thin polystyrene films. Eur. Phys. J. E 2008, 27, 375–377. 10.1140/epje/i2008-10394-3. 19030902

[ref33] Glor E. C.; Fakhraai Z. Facilitation of interfacial dynamics in entangled polymer films. J. Chem. Phys. 2014, 141, 194505 10.1063/1.4901512. 25416896

[ref34] Zhou Y.; Milner S. T. Short-Time Dynamics Reveals Tg Suppression in Simulated Polystyrene Thin Films. Macromolecules 2017, 50, 5599–5610. 10.1021/acs.macromol.7b00921.

[ref35] Zuo B.; Li C.; Xu Q.; Randazzo K.; Jiang N.; Wang X.; Priestley R. D. Ultrastable Glassy Polymer Films with an Ultradense Brush Morphology. ACS Nano 2021, 15, 9568–9576. 10.1021/acsnano.0c09631. 34032418

[ref36] Madkour S.; Gawek M.; Hertwig A.; Schonhals A. Do Interfacial Layers in Thin Films Act as an Independent Layer within Thin Films?. Macromolecules 2021, 54, 509–519. 10.1021/acs.macromol.0c02149.

[ref37] Press W. H.; Teukolsky S. A.; Vetterling W. T.; Flannery B. P. Numerical Recipes in C: The Art of Scientific Computing, 2nd ed.; Cambridge University Press: Cambridge, 1992.

[ref38] Gezerlis A. Numerical Methods in Physics with Python; Cambridge University Press: Cambridge, 2020.

[ref39] Mehta P.; Bukov M.; Wang C.-H.; Day A. G.; Richardson C.; Fisher C. K.; Schwab D. J. A high-bias, low-variance introduction to Machine Learning for physicists. Phys. Rep. 2019, 810, 1–124. 10.1016/j.physrep.2019.03.001. 31404441 PMC6688775

[ref40] van de Schoot R.; Depaoli S.; King R.; Kramer B.; Märtens K.; Tadesse M. G.; Vannucci M.; Gelman A.; Veen D.; Willemsen J.; Yau C. Bayesian statistics and modelling. Nature Reviews Methods Primers 2021, 1, 1 10.1038/s43586-020-00001-2.

[ref41] Betancourt M. A Conceptual Introduction to Hamiltonian Monte Carlo. arXiv 2017,1701.02434, DOI: 10.48550/arxiv.1701.02434.

[ref42] Abril-Pla O.; Andreani V.; Carroll C.; Dong L.; Fonnesbeck C. J.; Kochurov M.; Kumar R.; Lao J.; Luhmann C. C.; Martin O. A.; Osthege M.; Vieira R.; Wiecki T.; Zinkov R. PyMC: a modern, and comprehensive probabilistic programming framework in Python. PeerJ. Computer Science 2023, 9, e1516 10.7717/peerj-cs.1516. 37705656 PMC10495961

[ref43] Greiner R.; Schwarzl F. R. Thermal contraction and volume relaxation of amorphous polymers. Rheol. Acta 1984, 23, 378–395. 10.1007/BF01329190.

[ref44] Roth C. B.; Dutcher J. R. Glass transition and chain mobility in thin polymer films. J. Electroanal. Chem. 2005, 584, 13–22. 10.1016/j.jelechem.2004.03.003.

[ref45] Han Y.; Huang X.; Rohrbach A. C. W.; Roth C. B. Comparing refractive index and density changes with decreasing film thickness in thin supported films across different polymers. J. Chem. Phys. 2020, 153, 044902 10.1063/5.0012423. 32752678

[ref46] Barradas N. P.; Keddie J. L.; Sackin R. Bayesian inference analysis of ellipsometry data. Phys. Rev. E 1999, 59, 6138–6151. 10.1103/PhysRevE.59.6138. 11969599

[ref47] Hughes I. G.; Hase T. P. A. Measurements and Their Uncertainties; Oxford University Press: New York, 2010.

[ref48] Taylor J. R. An Introduction to Error Analysis: The Study of Uncertainties in Physical Measurements, 3rd ed.; University Science Books: New York, 2022.

[ref49] Ran R.; Pradeep S.; Kosgodagan Acharige S.; Blackwell B. C.; Kammer C.; Jerolmack D. J.; Arratia P. E. Understanding the rheology of kaolinite clay suspensions using Bayesian inference. J. Rheol. 2023, 67, 241–252. 10.1122/8.0000556.

[ref50] Banik I.; Pittordis C.; Sutherland W.; Famaey B.; Ibata R.; Mieske S.; Zhao H. Strong constraints on the gravitational law from Gaia DR3 wide binaries. Mon. Not. R. Astron. Soc. 2023, 527, 4573–4615. 10.1093/mnras/stad3393.

[ref51] Davidson-Pilon C. Bayesian Methods for Hackers: Probabilistic Programming and Bayesian Inference, 1st ed.; Addison-Wesley Professional, 2015.

[ref52] Frenkel D.; Smit B. Understanding Molecular Simulation: from Algorithms to Applications, 3rd ed.; Elsevier, 2023.

[ref53] Hoffman M. D.; Gelman A. The No-U-Turn sampler: Adaptively setting path lengths in Hamiltonian Monte Carlo. J. Mach. Learn. Res. 2014, 15, 1593–1623.

[ref54] Bittrich E.; Windrich F.; Martens D.; Bittrich L.; Häussler L.; Eichhorn K.-J. Determination of the glass transition temperature in thin polymeric films used for microelectronic packaging by temperature-dependent spectroscopic ellipsometry. Polym. Test. 2017, 64, 48–54. 10.1016/j.polymertesting.2017.09.030.

[ref55] Hajduk B.; Bednarski H.; Trzebicka B. Temperature-Dependent Spectroscopic Ellipsometry of Thin Polymer Films. J. Phys. Chem. B 2020, 124, 3229–3251. 10.1021/acs.jpcb.9b11863. 32275433 PMC7590969

[ref56] Yan J.; Xu J.; Weng L.-T.; Wang F.; Wang X.; Yuan H.; Wang T.; Tsui O. K. C. Glass Transition of the Surface Monolayer of Polystyrene Films with Different Film Thicknesses and Supporting Surfaces. Macromolecules 2023, 56, 556–566. 10.1021/acs.macromol.2c02013.

[ref57] Brandrup J.; Immergut E. H.; Grulke E. A.; Abe A.; Bloch D. R. Polymer Handbook; Wiley: New York, 1999; Vol. 89.

[ref58] Herminghaus S.; Jacobs K.; Seemann R. The glass transition of thin polymer films: some questions, and a possible answer. Eur. Phys. J. E 2001, 5, 531–538. 10.1007/s101890170036.

[ref59] Fujiwara H. Spectroscopic Ellipsometry: Principles and Applications; John Wiley & Sons, Ltd.: West Sussex, England, 2007.

[ref60] Ellison C. J.; Torkelson J. M. The distribution of glass-transition temperatures in nanoscopically confined glass formers. Nat. Mater. 2003, 2, 695–700. 10.1038/nmat980. 14502273

[ref61] van Zanten J. H.; Wallace W. E.; Wu W.-L. Effect of strongly favorable substrate interactions on the thermal properties of ultrathin polymer films. Phys. Rev. E 1996, 53, R2053–R2056. 10.1103/PhysRevE.53.R2053. 9964605

[ref62] Park C. H.; Kim J. H.; Ree M.; Sohn B.-H.; Jung J. C.; Zin W.-C. Thickness and composition dependence of the glass transition temperature in thin random copolymer films. Polymer 2004, 45, 4507–4513. 10.1016/j.polymer.2004.04.048.

[ref63] Roth C. B.; McNerny K. L.; Jager W. F.; Torkelson J. M. Eliminating the enhanced mobility at the free surface of polystyrene: Fluorescence studies of the glass transition temperature in thin bilayer films of immiscible polymers. Macromolecules 2007, 40, 2568–2574. 10.1021/ma062864w.

[ref64] Madkour S.; Yin H.; Füllbrandt M.; Schönhals A. Calorimetric evidence for a mobile surface layer in ultrathin polymeric films: poly(2-vinyl pyridine). Soft Matter 2015, 11, 7942–7952. 10.1039/C5SM01558H. 26324951

[ref65] Zhang J.; Ji H.; Yang J.; Huang J. Two glass transitions in substrate-supported polymer ultrathin films: A molecular dynamics simulation. Polymer 2024, 308, 127410 10.1016/j.polymer.2024.127410.

[ref66] Han Y.; Roth C. B. Gradient in refractive index reveals denser near free surface region in thin polymer films. J. Chem. Phys. 2021, 155, 144901 10.1063/5.0062054. 34654302

[ref67] Paeng K.; Richert R.; Ediger M. D. Molecular mobility in supported thin films of polystyrene, poly(methyl methacrylate), and poly(2-vinyl pyridine) probed by dye reorientation. Soft Matter 2012, 8, 819–826. 10.1039/C1SM06501G.

[ref68] Holt A. P.; Griffin P. J.; Bocharova V.; Agapov A. L.; Imel A. E.; Dadmun M. D.; Sangoro J. R.; Sokolov A. P. Dynamics at the Polymer/Nanoparticle Interface in Poly(2-vinylpyridine)/Silica Nanocomposites. Macromolecules 2014, 47, 1837–1843. 10.1021/ma5000317.

