# Supplementary material for: A Bayesian Inference Approach to Accurately Fitting the Glass Transition Temperature in Thin Polymer Films
Source: Macromolecules. 2024 Nov 22;57(23):11055–74. doi: 10.1021/acs.macromol.4c01867 (PMC11636260; doi:10.1021/acs.macromol.4c01867)
Supplement: Supplementary file 1 — ma4c01867_si_001.pdf [file ma4c01867_si_001.pdf]

SUPPORTING INFORMATION

A Bayesian Inference Approach to Accurately Fitting the Glass Transition Temperature in Thin Polymer Films

James H. Merrill, Yixuan Han, and Connie B. Roth*

Department of Physics, Emory University, Atlanta, Georgia, 30322 USA

*To whom correspondence should be should be addressed. Email: cbroth@emory.edu

jmerri8 / PyTg

Code Issues Pull requests Actions Projects Wiki Security

PyTg / README.md

jmerri8 Update README.md 12164e5 · 2 hours ago

51 lines (39 loc) · 4.02 KB

Preview Code Blame Raw

PyTg

PyTg is a set of scripts for performing Bayesian inference fits on ellipsometric measurements of the glass transition temperature. In order to run the scripts, a working installation of Python with PyMC and its dependencies is required. We recommend first installing Anaconda python (available at https://www.anaconda.com/download) to streamline this process. A virtual python installation environment is preferred (see https://www.pymc.io/projects/docs/en/stable/installation.html) to preserve the state of any existing base python environment on your machine. Running "env_setup.py" will do this automatically when run from the Anaconda prompt.

Function definitions

bayes_fit(fname, draw=4000, tune=2000, plot_ppc = True, save_data = True, save_summary = True, return_data = True, fit_error = True):

This function takes a filename for a dataset to fit, a number of MC samples to draw after the NUTS tuning steps, a number of MC steps to use for tuning NUTS parameters, a boolean variable for whether to plot the posterior predictive distribution, a boolean variable for whether to save the resulting `InferenceData` object to a file, a boolean variable for whether to save the resulting text fit summary to a file, a boolean variable for whether to return the data that was fit as a numpy array, and a boolean variable for whether to use the supplied errors to weight the fits, or fit the noise in the data as a parameter in the model. It returns an ArviZ `InferenceData` object containing the model, parameters, and all of the MC sampling data. Optionally, it can return the data that was fit (useful for customizing graphs). Example usage is provided in the main script.

Parameters

```
fname : filename or file handle
    Filename for the dataset to fit. Requirements: 3-column .txt file,
with 1 line of header and no footer. Independent variable (x) in the
first column, dependent variable in the second column, and error in the
dependent variable in the third column.
draw : int, optional
    The number of samples to draw. The default is 2000.
tune : int, optional
    Number of samples to use for tuning the adaptive step length for
NUTS. The default is 1000.
plot_ppc : bool, optional
    Choose whether the posterior predictive distribution, which
generates simulated data from the posterior distributions of each param
in the model, should be sampled and plotted. The default is True.
save_data : bool, optional
    Choose whether to save the inference data generated by pymc. The
default is True.
save_summary : bool, optional
    Choose whether to save the text summary of the inference performed.
The default is True.
return_data : bool, optional
    Choose whether to return the raw data that was fit. The default is
True.
fit_error : bool, optional
    Choose whether to fit the noise in the data as a parameter, or not.
```

```
The default is False. WARNING: SETTING TO TRUE WILL MAKE ABSOLUTE
VALUES OF FIT PARAMETER ERRORS UNPHYSICAL!
```

Returns

```
trace : pymc.backends.base.MultiTrace or arviz.InferenceData
    A ``MultiTrace`` or ArviZ ``InferenceData`` object that contains
the samples.
count_data : array-like, optional
    Unscaled film thickness or intensity
T : array-like, optional
    Unscaled temperature array
```

extract_values(trace, parameter):

This function takes an InferenceData object, and the name of a parameter (as a string) in the model inside that inference object to extract as an array. It returns the specified variable, with all sampled values of that parameter, as a numpy array. Example usage is provided in the main script.

Parameters

```
trace : InferenceData
    The InferenceData object containing the sampled values of interest.
parameter : String
    Name of the model parameter inside the InferenceData object to be
extracted.
```

Returns

```
params : Numpy Array
    Returns 1D array of all values taken on by the specified parameter
during MCMC sampling.
```

Link to github: https://github.com/jmerri8/PyTg

```python
#!/usr/bin/env python3
# -*- coding: utf-8 -*-

"""
Created on Thu Nov 11 12:16:29 2021

@author: James H. Merrill, Department of Physics, Emory University
"""


from IPython.core.pylabtools import figsize
import numpy as np
from matplotlib import pyplot as plt
from tkinter import *
from tkinter import filedialog
import pymc as pm
import arviz as az
import scipy.stats as stats
import xarray as xr
import time as t
import pandas as pd
import sys
import os


def bayes_fit(fname, draw=4000, tune=2000, plot_ppc = True, save_data = True,
              save_summary = True, return_data = True, fit_error = True):
    """


    Parameters
    ----------
    fname : filename or file handle
        Filename for the dataset to fit. Required formatting: 3 column, tab-delimited plaintext,
        one row of header and no footer. Columns must contain T, h, h_err in that order.
    draw : int, optional
        The number of samples to draw from the posterior distribution. The default is 4000.
    tune : int, optional
        Number of samples to use for tuning the adaptive step length for NUTS. The default is 2000.
    plot_ppc : bool, optional
        Choose whether the posterior predictive distribution, which generates simulated data from
        the posterior distributions of each param in the model, should be sampled and plotted.
        The default is True.
    save_data : bool, optional
        Choose whether to save the inference data generated by pymc. The default is True.
    save_summary : bool, optional
        Choose whether to save the text summary of the inference performed. The default is True.
    return_data : bool, optional
        Choose whether to return the raw data that was fit.
    fit_error : bool, optional
        Choose whether to fit the noise in the data as a parameter, or not. The default is False.
        WARNING: SETTING TO TRUE WILL MAKE ABSOLUTE VALUES OF FIT PARAMETER ERRORS UNPHYSICAL.
    Returns
    -------
```

```
    trace : pymc.backends.base.MultiTrace or arviz.InferenceData
        A ``MultiTrace`` or ArviZ ``InferenceData`` object that contains the samples.
    count_data : array-like, optional
        Unscaled film thickness array
    T : array-like, optional
        Unscaled temperature array

    """
    dat = np.loadtxt(fname, skiprows = 1, unpack = False)
    count_data = dat.transpose()[1]
    T = dat.transpose()[0]
    h_err = dat.transpose()[2]
    target_accept = 0.8 #same as default value for NUTS


    n_count_data = len(count_data)
    x_n = stats.zscore(T) #standardize T
    y = count_data
    y_n = stats.zscore(y) # standardize h
    with pm.Model() as model:

        #define priors: initial guess for shape and initial values for probability
        #distributions of each variable in model
        if fit_error is False:
            sigma = np.mean(h_err)/np.std(y)
        else:
            sigma = pm.HalfCauchy("Sigma", beta = .1)
        intercept = pm.Normal("Intercept", mu=0, sigma=1)
        M = pm.Normal("Melt linear expansion coefficient", mu=0, sigma=1)
        G = pm.Normal("Glassy linear expansion coefficient", mu=0, sigma =1)
        w = pm.HalfNormal("Transition width")

        tg = pm.Uniform('Tg', lower = min(x_n), upper = max(x_n))
        # ~uniform probability over length of temperature array
        # tg = pm.Normal('Tg', mu = 1, sigma=1)


        h_T = (w * (M-G) /2.0 *  pm.math.log(pm.math.cosh((x_n-tg)/w))+(x_n-tg)*(M+G)/2.0

               + intercept)

        y_obs = pm.Normal("y_obs", mu = h_T, sigma = sigma, observed=y_n) #likelihood
        trace = pm.sample(draws=draw, tune=tune, init = 'jitter + adapt_diag',
                          step=pm.NUTS(target_accept = target_accept), cores=4,
                          progressbar=TRUE)
        pp = pm.sampling.sample_posterior_predictive(trace, keep_size = True,
                                                     extend_inferencedata = True)
        prior = pm.sample_prior_predictive(samples=n_count_data)
        az.InferenceData.extend(trace, prior)


        if(plot_ppc is True):
            #generate posterior predictive samples and extend 'trace' with these samples
            pp = pm.sampling.sample_posterior_predictive(trace, keep_size = True,
                                                         extend_inferencedata = True)
            prior = pm.sample_prior_predictive(samples=n_count_data)
            az.InferenceData.extend(trace, prior) #add prior predictive data to trace
            az.plot_ppc(pp, 'cumulative', alpha = .2) #cumulative prob. density for y_obs
            az.plot_ppc(pp) #plot the pdf of y_obs
            az.plot_lm(y = trace.observed_data["y_obs"],
                       idata = trace,
                       y_hat = trace.posterior_predictive["y_obs"], x= T,
                       legend = True, grid = False, figsize = (8,9),
                       textsize = 14)
        summary = az.summary(trace, round_to = 4)
        print(summary)
        now = str(t.ctime())
```

```
        if save_data is True:
            np.save("inference_data_" + now, trace)
        if save_summary is True:
            np.save("inference_summary_" +now, summary)
        if return_data is True:
            return trace, count_data, T, h_err
        else:
            return trace


trace, h, T, h_err = bayes_fit(fname = filedialog.askopenfilename(multiple = False),
                               draw = 2000,
                               tune = 2000,
                               save_data = False,
                               save_summary = False,
                               plot_ppc = True,
                               fit_error = False)
#uncomment line below to show traceplot (parameter value histograms, and
#value vs. iteration # for each param)
#az.plot_trace(trace, figsize=(12, 36))

#calculate predicted and true values of the dependent variable in the model (e.g., h)
# to compute effective r-squared
y_pred = trace.posterior_predictive.stack(sample=("chain", "draw"))["y_obs"].values.T
y_true = trace.observed_data["y_obs"].values.T

print(az.r2_score(y_true, y_pred))
print("h ~ "+ str(min(h)))


def extract_values(trace, parameter):
    """
    Parameters
    ----------
    trace : InferenceData
        The InferenceData object containing the sampled values of interest.
    parameter : String
        Name of the model parameter inside the InferenceData object to be extracted.

    Returns
    -------
    params : Numpy Array
        Returns 1D array of all values taken on by the specified parameter during MCMC sampling.

    """
    param_arr =  trace.posterior[parameter].values
    params = []
    for chain in param_arr:
        for param in chain:
            params.append(param)
    params = np.array(params)
    return params

tgvals = extract_values(trace, "Tg")
tgvals = tgvals * np.std(T) + np.mean(T)  #convert to real units
Mvals = extract_values(trace, "Melt linear expansion coefficient")
Gvals = extract_values(trace, "Glassy linear expansion coefficient")
wvals = extract_values(trace, "Transition width") * np.std(T) #convert to real units
hvals = extract_values(trace, "Intercept") *np.std(h)+np.mean(h) #convert to real units

TgErr = np.std(tgvals)

alpha_Mvals = Mvals*np.std(h)/np.std(T)/hvals.mean() #convert to real units
alpha_Gvals = Gvals*np.std(h)/np.std(T)/hvals.mean() #convert to real units

alpha_M = np.mean(alpha_Mvals)
```

```
alpha_M_err = np.std(alpha_Mvals)
alpha_G = np.mean(alpha_Gvals)
alpha_G_err = np.std(alpha_Gvals)
alphas = [alpha_G, alpha_G_err, alpha_M, alpha_M_err]
report_string = ""
# for x in alphas:
#     report_string += (str(x) + "\t")
# print("Tg, Tgerr = " + str(np.mean(tgvals)) + "\t" + str(np.std(tgvals)) )
# print("alpha_G, alpha_G err, alpha_M, alpha_M err = " + report_string)


```
